# Bicalutamide Elicits Renal Damage by Causing Mitochondrial Dysfunction via ROS Damage and Upregulation of HIF-1α

**DOI:** 10.3390/ijms21093400

**Published:** 2020-05-11

**Authors:** Kuan-Chou Chen, Chang-Rong Chen, Chang-Yu Chen, Kai-Yi Tzou, Chiung-Chi Peng, Robert Y. Peng

**Affiliations:** 1 Graduate Institute of Clinical Medicine, College of Medicine, Taipei Medical University, Taipei 11031, Taiwan; kuanchou@tmu.edu.tw (K.-C.C.); kaietzou@gmail.com (K.-Y.T.); 2 Department of Urology, School of Medicine, College of Medicine, Taipei Medical University, Taipei 11031, Taiwan; 3 Department of Urology, Taipei Medical University Shuang-Ho Hospital, New Taipei City 23561, Taiwan; 4 TMU-Research Center of Urology and Kidney, Taipei Medical University, Taipei 11031, Taiwan; 5 International Medical Doctor Program, Vita-Salute San Raffaele University, 20132 Milan, Italy; cherylcherylchen@gmail.com; 6 Program of Biomedical Sciences, College of Arts and Sciences, California Baptist University, Riverside, CA 92504, USA; eugenechen0529@gmail.com; 7 Department of Biotechnology, College of Medical and Health Care, Hungkuang University, Shalu District, Taichung 43302, Taiwan; ypeng@seed.net.tw

**Keywords:** bicalutamide (Bic), rat mesangial cell (RMC) line, mitochondrial dysfunction, HIF-1α, oxygen consumption rate (OCR)

## Abstract

Combined androgen blockade using bicalutamide (Bic) is a therapeutic choice for treating prostate cancer (PCa). However, even at regular clinical dosages, Bic frequently shows adverse effects associated with cardiovascular and renal damage. Previously, we found that Bic selectively damaged mesangial cells compared to tubular cells and in an in vivo rat model, we also found renal damage caused by Bic. In the present study, a rat mesangial cell model was used to further the investigation. Results indicated that Bic enhanced lactate dehydrogenase release, reactive oxygen species (ROS) production, lysosome population and kidney injury molecule-1 and decreased N-cadherin. Bic elicited mitochondrial swelling and reduced the mitochondrial potential, resulting in severe suppression of the oxygen consumption rate (OCR), maximum respiration and ATP production. The hypoxia-inducible factor (HIF)-1α transcriptional activity and messenger RNA were significantly upregulated in dose-dependent manners. The HIF-1α protein reached a peak value at 24 h then rapidly decayed. BCL2/adenovirus E1B 19-kDa protein-interacting protein 3 and cleaved caspase-3 were dose-dependently upregulated by Bic (60 μM) and that eventually led to cell apoptosis. It is suggested that Bic induces renal damage via ROS and modulates HIF-1α pathway and clinically, some protective agents like antioxidants are recommended for co-treatment.

## 1. Introduction

Bicalutamide (Bic) is a non-steroidal antiandrogen which provides blockade at tumor sites to facilitate treatment of prostate cancer (PCa) [1]. Bic possesses excellent affinities to androgen receptors [2], through which Bic induces apoptosis of androgen-dependent benign PWR-1E prostatic cells. However, in androgen-independent PC-3 cells, Bic also induces apoptosis by mechanisms partially inhibited by pan-caspase inhibition [3].

Although Bic is deemed to be “nontoxic” and “harmless” [4], a diversity of studies are emerging pointing to multiple organ injuries after its use, including hepatotoxicity [5,6,7,8], cardiovascular diseases (CVDs, frequency 7% with 150 mg Bic) [9,10] and many others [11,12]. A report from the US Food and Drug Administration demonstrated that up to 37.5% of patients who have taken Bic therapy for 1–6 months may experience kidney failure, with an incidence rate of 94.17% for males aged over 60 years [13]. Recently, the literature also revealed that Bic and leuprorelin induced tubulointerstitial nephritis, interstitial pneumonitis and liver dysfunction [6]. Previously, we also recognized a Bic-increased renal-damaging effect in vitro and in vivo (Figure S1) [14].

Overall mechanisms of the adverse effects on organs by Bic are not well established. Some speculations indicated that Bic-induced liver injury might be due to its toxic metabolites [15]. Otherwise, oxidative stress and mitochondrial toxicity might also play important roles [16,17]. To date, straightforward evidence showing the renal-damaging effect of Bic therapy is still lacking.

Studies of men undergoing long-term (≥12 months) combined androgen blockade (CAB) revealed higher prevalence of diabetes mellitus (DM) and metabolic syndrome compared to controls [18,19]. Administration of leuprolide and bicalutamide significantly decreased insulin sensitivity, implying the potential of androgen-deprivation therapy (ADT) to elicit DM [20]. An increased degree of insulin resistance (IR) can trigger progressive renal function decline via a complex mechanism [21]. Etiologically, IR was implicated in the development of glomerular hypertension and hyperfiltration [22], as usually found in the initial phase of diabetic kidney disease [23,24]. As is well known, metabolic and hemodynamic perturbations interact and play critical roles in the pathophysiological mechanisms leading to kidney disease progression [23,25,26]. Diabetes alone has been implicated as an independent risk factor for acute kidney injury (AKI), particularly when some caustic medications are consumed [27].

Alternately, Bic may exert apoptotic effects independent of its antiandrogenic ability. Bic decreases prostatic blood flow and induces apoptosis via a hypoxic pathway [28]. As usual, the cellular response to hypoxia is focused on hypoxia-inducible factors (HIFs) and HIF was demonstrated to be initially activated to attenuate renal injury in a chronic kidney disease (CKD) model of a subtotal nephrectomy [29,30]. Much of the literature has implicated chronic hypoxia as a common final pathway leading to the development of end-stage renal failure [31,32]. Hypoxia decreases the mitochondrial membrane potential (MMP) and suppresses the mitochondrial respiratory chain function, resulting in apoptosis of glomerular endothelial cells and a failure of tissue oxygenation following kidney injury [33,34,35], thereby stimulating transforming growth factor (TGF)-β-mediated fibroblast activation and contributing to renal fibrosis [36]. It is worth noting that hypoxia-related genes, including cyclin G2, BCL2-interacting protein 3 (BNIP3) and glucose transporter (Glut)-1, were shown to be highly expressed in LNCaP cells treated with Bic [37]. And more importantly, mitochondrial dysfunction gives rise to CKD progression irrespective of underlying causes [38].

Considering that mitochondria play a critical role in cellular energy production and this phenomenon has gained much attention for its opposing roles in cell survival and cell death [39], we propose that Bic might be able to evoke renal injury via lowering oxygen tension and damaging mitochondria. We performed this study to reveal such a relevant effect.

## 2. Results and Discussion

### 2.1. Lactate Dehydrogenase (LDH) and Kidney Injury Molecule (KIM)-1 Are Affected by Bic in RMC Cells

**Figure 1:**
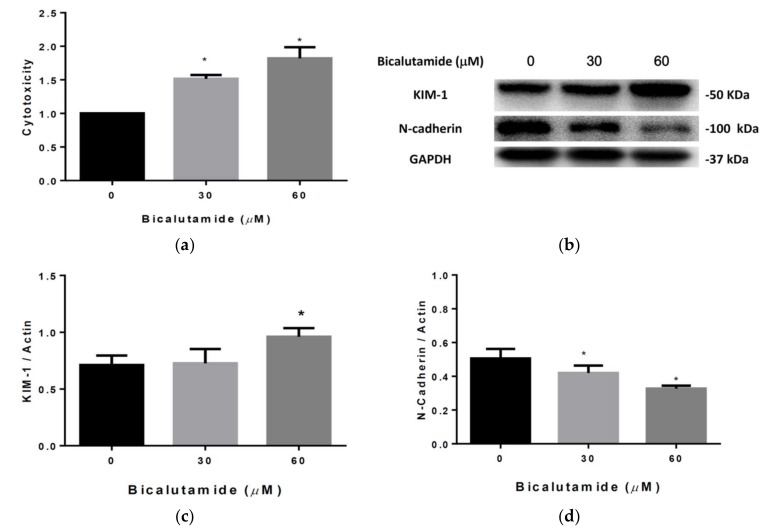
Lactate dehydrogenase (LDH) assay and the Western blot of kidney injury molecule (KIM)-1 and N-cadherin in rat mesangial cells (RMCs). Cells were treated with bicalutamide (Bic) at concentrations of 30 and 60 μM at 24 h. (**a**) LDH release was increased by Bic (*n* = 3, * *p* < 0.05). (**b**) A representative blot of protein expressions of KIM-1 and N-cadherin. GAPDH was used as an internal control. (**c**) Quantitative data of Western blotting of KIM-1(*n* = 3, * *p* < 0.05). (**d**) Quantitative data of Western blotting of N-cadherin (*n* = 3, * *p* < 0.05). When RMCs were treated with Bic, N-cadherin dose-dependently decreased, however KIM-1 was significantly induced in the group treated with 60 μM.

LDH release was induced by Bic at 24 h in a dose-dependent manner (Figure 1a). The elevation of enzyme activity had reached 1.5- and 1.8-fold compared to the controls (Figure 1a). LDH is a soluble cytoplasmic enzyme present in almost all cells and is released into the extracellular space when plasma membranes are damaged [40]. LDH activity was shown to be elevated in chronic renal failure and was also correlated with the blood urea nitrogen (BUN) level [41]. KIM-1 is a type 1 transmembrane protein, which is not detectable in normal human and rodent kidneys but is markedly upregulated in acute tubular necrosis [42,43]. KIM-1 is also a potential biomarker for glomerular injury in proteinuric kidney disease [44]. N-Cadherin is expressed in multiple tissues and acts to mediate cell-cell adhesion. In mesangial cells, N-cadherin plays a role in counteracting tensional forces generated in glomerular capillary walls [45]. Some literature is emerging suggesting that loss of cadherins is a critical initial step in eliciting progressive kidney disease and further stimulating cell migration [46,47], as evidenced by many studies [48]. In clinical prescriptions, a regular dose of Bic is 30–60 μM, which we showed to have caused significant LDH release and KIM-1 upregulation (Figure 1a–c) at 24 h and to have simultaneously downregulated N-cadherin (Figure 1b, 1d) in our cell model, implying the probability that Bic at a dose of ≥60 μM might induce AKI.

It is worth mentioning that in addition to the biomarkers of KIM-1 and N-cadherin, neutrophil gelatinase-associated lipocalin (NGAL) is a very useful biomarker widely expressed in a variety of cell types, including neutrophils, mesangial cells and tubular cells [49,50]. NGAL is upregulated in resident cells in response to renal injury, as demonstrated in patients with acute nephrotoxicity or proliferative glomerulonephritis [51]. The severity of kidney injury and sensitivity of NGAL have been applied translationally, where serum and urine NGAL levels were successfully used for non-invasive assessments of renal damage in increasing numbers of clinical conditions [49,50] and this is worth evaluating in our future research work.

### 2.2. Oxidative Stress Induced by Bic in RMCs Is Dose-dependent

**Figure 2:**
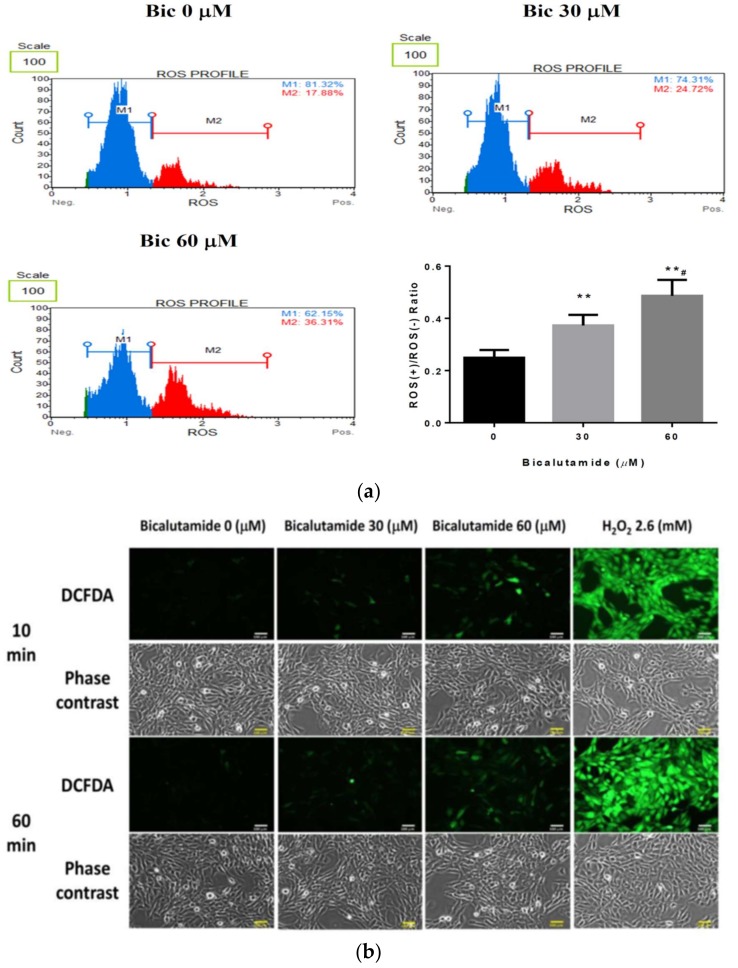
Measurement of oxidative stress. Reactive oxygen species (ROS) production induced by bicalutamide (Bic) was measured by (**a**) dihydroethidium (DHE) flow cytometry at 60 min and (**b**) dichlorodihydrofluorescein diacetate (DCFDA) staining at 10 and 60 min (^#^ *p* < 0.05; ** *p* < 0.01; Scale bar=100 μM). Bic dose-dependently induced ROS production, as shown by DHE flow cytometry and DCFDA fluorescence staining. Data are expressed as the mean±standard deviation (*n* = 3).

Of all cellular ROS sources, electron leakage from the mitochondrial electron transfer chain to molecular oxygen generates a steady flux of superoxide anion (^●^O_2_^−^) and thus constitutes a major site of cellular ROS production [52,53]. Dihydroethidium (DHE) is known to be the most specific fluorescent probe for superoxide detection [54]. After treatment with 30 and 60 μM Bic for 1 h, the percentage of ethidium-positive cells was seen to increase in a dose-dependent manner, at proportions of 36% and 51%, respectively, compared to 23% in the control group (Figure 2a). 2′, 7–dichlorofluorescin diacetate (DCFDA) fluorescence is triggered by oxidation via hydrogen peroxides and hydroxyl radicals [55]. Bic induced free radicals and also non-radicals of ROS production, as revealed by the intensity of fluorescence in time- (10–60 min) and dose-dependent (0–60 μM) manners (Figure 2b) and the cell density was also likely correspondingly reduced (Figure 2b). A significant increase in oxidative stress was described in Bic-treated PCa cells; thus oxidative stress and apoptosis via caspase-3 activation are key executioners in caspase-mediated cell death [56]. 

### 2.3. Mitochondrial Deterioration Affected by Bic in RMCs

In healthy cells with a high mitochondrial potential (Δψ_M_), JC-1 spontaneously forms J-aggregates with emission of intense red fluorescence (fluorescence emission at ~590 nm). While in apoptotic or unhealthy cells with a low Δψ_M_, JC-1 shows only green fluorescence (fluorescence emission at ~529 nm) [57]. Consequently, JC-1 is widely used in apoptosis studies to monitor mitochondrial health [57].

**Figure 3:**
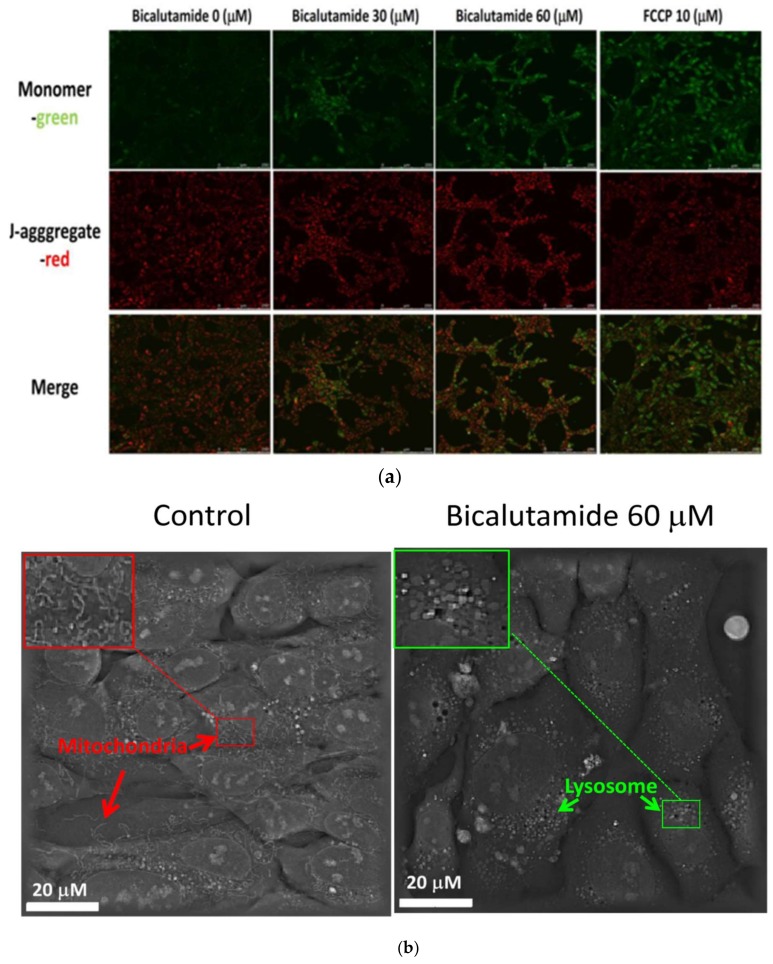

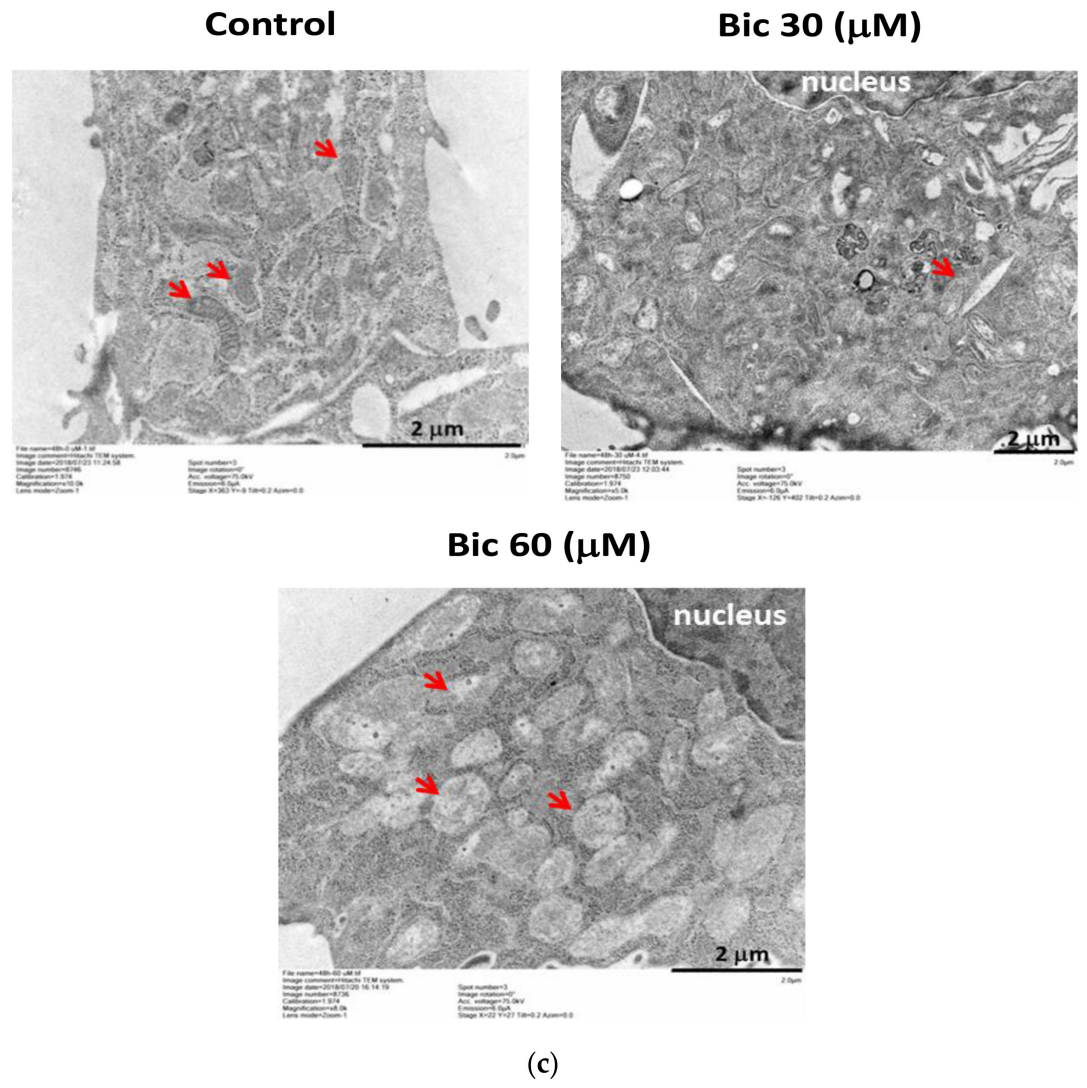
Estimation of the mitochondrial membrane potential and morphological changes. (**a**) Cells were treated with bicalutamide (Bic) at 30 and 60 μM for 24 h. JC-1 staining showed changes in the mitochondrial potential affected by Bic therapy. Scale bar = 250 μM (**b**) Living cell tomographic microscopy of rat mesangial cells (RMCs) affected by Bic (60 μM) at 48 h as viewed with Nanolive 3D cell explorer (microscale length: 20 μm, magnification: × 400). Control images revealed mitochondria containing many fibrous structures (indicated by a red arrow, also see enlarged insert), which were less seen in Bic (60 μM)-treated cells and clusters of lysosomes instead appeared in treated cells (indicated by a green arrow, also see enlarged insert). (**c**) Morphological changes in mitochondria after being treated with Bic at 30 and 60 μM for 48 h. As seen, the mitochondria treated with 30 μM Bic were only slightly swollen, while those treated with 60 μM were severely swollen (
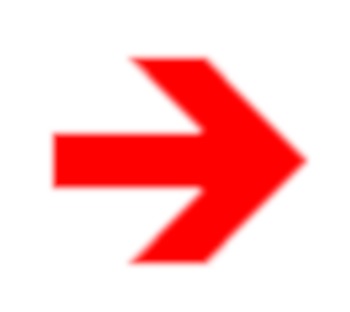
red arrow: mitochondria). Scale bar = 2 μm.

As can obviously be seen, in the control group, the content of red J-aggregate prevailed, while the aggregates decreased and green monomers dose-dependently increased with Bic at 24 h, implying a decreasing effect of Bic on the membrane potential (Δψ_M_) (Figure 3a). Bic induced apoptosis by depolarization of the MMP in the PC-3 PCa cell line [58]. In parallel, FCCP, a protonophore that can depolarize mitochondrial membranes, was added as a positive control for JC-1 staining [59]. We found that most green fluorescence appeared in RMCs after treatment with FCCP (10 μM) for 1 h (Figure 3a). Mitochondrial oxidative phosphorylation (OXPHOS) plays a central role in ATP production. Renal tissues are highly dependent on oxygen and are especially susceptible to a defective OXPHOS status, which in turn may decrease Δψ_M_ for ATP synthesis in a variety of kidney diseases [60]. An in vivo 5/6 nephrectomy CKD model displayed marked mitochondrial dysfunction with decreases in the MMP, ATP production and mitochondrial (mt)DNA copy number and an increase in mitochondrial ROS in renal tissues [61]. Consistent with this, under a 3D live microscope, it was found that in RMCs treated with 60 μM Bic, the mitochondrial mass obviously decreased and instead, more lysosomes appeared at 48 h (Figure 3b). A vast number of pathological conditions are associated with imbalances in the mitochondrial mass [62]. It is known that mitochondria respond to stress by attempting to maintain their structure and composition [63]. Damaged mitochondria are identified by autophagosomes and are subsequently degraded by lysosomes [63]. In an animal model of experimental proteinuria, activities of lysosomal enzymes in the renal cortex and urine were significantly higher in proteinuric compared to non-proteinuric rats [64]. Previously, Widlansky et al.’s study recognized such a phenomenon in mononuclear cells of type 2 diabetes mellitus (T2DM) patients [65]. It was proposed that Bic induced mitochondrial damage in RMCs, which may worsen the progression of diabetic kidney disease.

Normally in most eukaryotic cells, the double-membrane structure of mitochondria constitutes three divided regions and two compartments, designated the outer mitochondrial membrane (OMM), the intermembrane space, the inner mitochondrial membrane (IMM) that forms cristae and the matrix [66]. Cristae harbor respiratory chain complexes that are embedded within and peripheral to the membrane, forming a tightly organized unit critical for efficient electron transfer via the OXPHOS system [67], which however can be further modulated by morphological changes in the IMM. In untreated RMCs, there was an immense population of elongated mitochondria with the clear appearance of cristae (Figure 3c). In contrast, when RMCs were treated with Bic at 30 or 60 μM for 48 h, most of the mitochondria were damaged, appearing moderately or severely swollen and lacking cristae. Importantly, these ultrastructural changes well coincided with a tendency of a decrease in the membrane potential (Figure 3a, c). Mitochondrial swelling might be an early sign of apoptosis [68]. Disturbance of cristae may affect the structure of the OXPHOS system, which impairs cellular metabolism and growth [67].

Controversially, a study by Ball et al (2016) reported that ‘Bicalutamide was not found to be a mitochondrial toxicant” and we suspect that different cell lines have different susceptibilities [69]. Ball et al. used HepG2 cells, while ours were RMCs. The former cells are like liver cells, while the latter are of renal cell origin and there must be some tissue-specific tolerance and susceptibility [69].

### 2.4. Bioenergetic Profile of RMCs Affected by Bic

**Figure 4:**
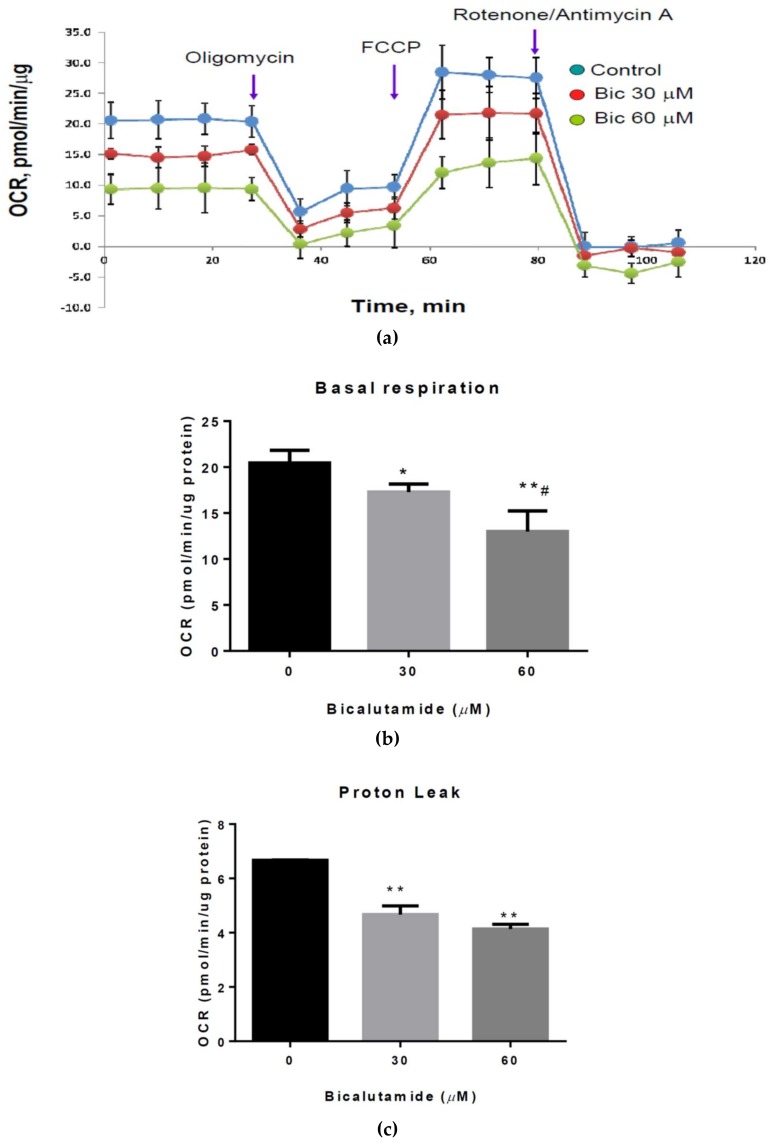

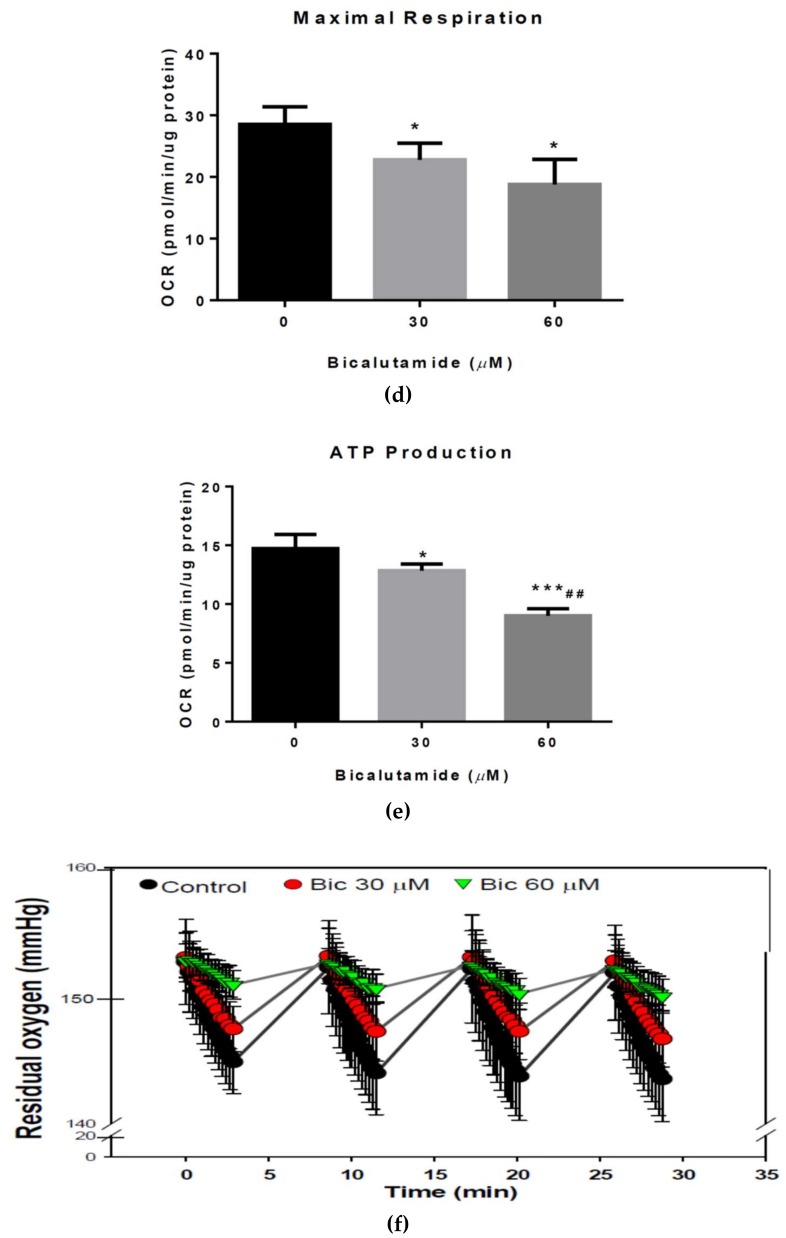
Parameters of the mitochondrial oxygen consumption rate (OCR) and residual oxygen affected by bicalutamide (Bic). Rat mesangial cells (RMCs) were untreated or treated with Bic at doses of 30 and 60 μM for 24 h. The basal respiration, maximal respiration and ATP production in cells all decreased. More residual oxygen consumption (ROX) remained at the higher dosage of Bic. (**a**) Time course and OCR curve plot obtained by a Seahorse analyzer. Quantification of changes in parameters induced by Bic treatment included (**b**) basal respiration, (**c**) proton leakage, (**d**) maximal respiration, (**e**) ATP production and (**f**) time course of residual oxygen in RMCs untreated or treated with Bic at 30 and 60 μM. Data are expressed as the mean±standard deviation (*n* = 3). * *p* < 0.05, ** *p* < 0.01, *** *p* < 0.005 compared to untreated cells; ^#^ *p* < 0.05, ^##^ *p* < 0.01 compared to 30 μM Bic-treated cells.

As the MMP was altered, we next used an Agilent Seahorse XFe96 Analyzer to determine whether Bic affects mitochondrial function and the bioenergenesis of RMCs. Normally, intracellular substrate oxidation produces ATP at the expense of oxygen consumption. The entire electron transport chain is predominantly controlled by parallel re-entry pathways through ATP synthase and proton leakage [70]. The addition of mitochondrial inhibitors such as a combination of rotenone (an inhibitor of complex I) and antimycin (an inhibitor of complex III) can completely shut down the electron transport chain even when complex V is already inhibited [71]. The entire range of the mitochondrial OCR measured by the Seahorse assay is shown in Figure 4a. Respective ranges of the OCR affected by the control and Bic at 30 and 60 μM were: basal respiration (20.4 ± 2.0, 17.3 ± 1.8 and 12.9 ± 4.0 pmol/min/μg protein), proton leakage (6.7 ± 1.0, 4.7 ± 1.1 and 4.1 ± 1.0 pmol/min/μg protein), maximal respiration (28.5 ± 3.0, 22.8 ± 3.0 and 18.8 ± 5.0 pmol/min/μg protein) and ATP production (14.7 ± 5.2, 12.9 ± 1.0 and 9.0 ± 1.0 pmol/min/μg protein) (Figure 4b–e). The basal respiration, maximal respiration and ATP production in RMCs were all significantly suppressed by treatment with Bic (60 μM) for 24 h (*p* < 0.05) (Figure 4b–e). Consistent with our results, Ming et al. indicated that a clinically relevant dose of Bic (2 mg/kg/day) (for a 70 kg male, about 140 mg per day) decreased tumor oxygenation by 45% within 24 h [72]. A rather interesting phenomenon reported by Pignatta et al. demonstrated that prolonged exposure to Bic therapy may generate an androgen-independent phenotype with alteration of mitochondrial dynamics and breakdown in the Drp-1-mediated mitochondrial network [73]. Recent studies demonstrated that targeting the phosphatidylinositide 3-kinase (PI3K)/AKT signaling pathway is a major strategy for treating androgen-independent PCa [74] or alternately meclofenamic acid can be prescribed. Meclofenamic acid is a nonsteroidal anti-inflammatory drug that has shown therapeutic potential for different types of cancers, including androgen-independent PCa [75]. The time point for intervention application was suggested to be at the point when the level of prostate-specific antigen retrogrades.

When electron transport chain inhibitors are added, residual oxygen consumption (ROX) is due only to non-mitochondrial respiration caused by some oxidases and other cellular enzymes [69]. ROX was increased by Bic therapy in a dose-dependent manner (Figure 4f). As seen, after treatment with 30 μM Bic, the value remained high at 145.5 mmHg until 20 min but declined to 143.2 mmHg at 27 min. A higher dose (60 μM) of Bic yielded a higher ROX, which was suppressed from 151 mmHg (at 3 min) to 150 mmHg at 27 min (Figure 4f).

### 2.5. Bic Increased HIF-1α Transcriptional Activity and HIF-1α mRNA, Resulting in Accumulation of the HIF-1α Protein

**Figure 5:**
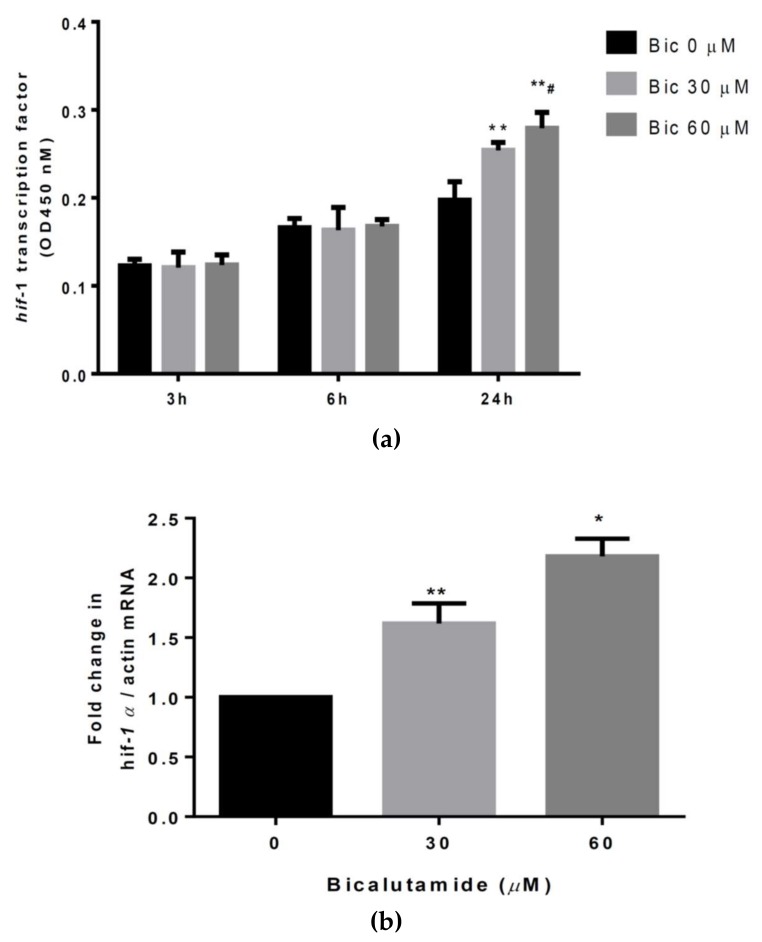

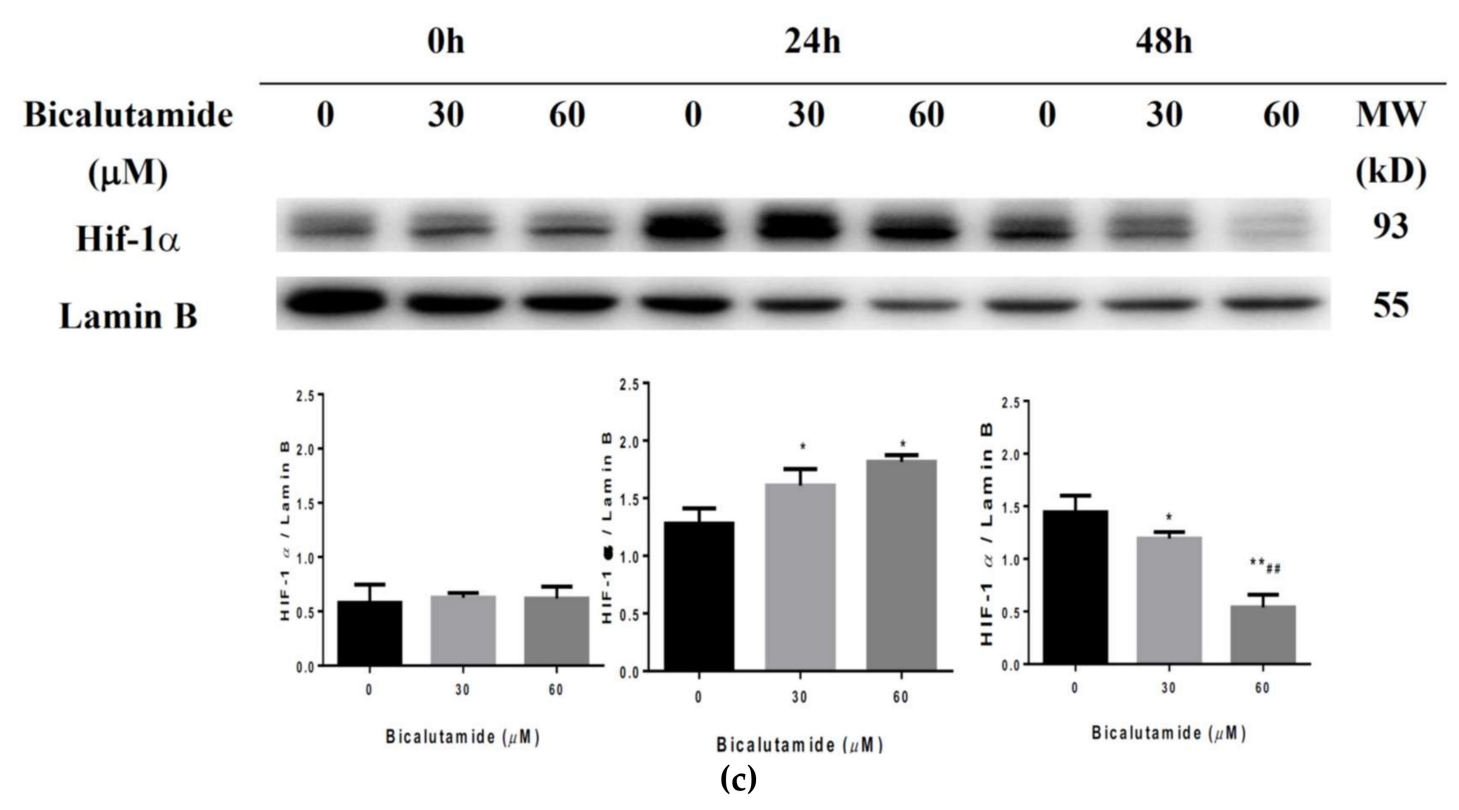
(**a**) Hypoxia-inducible factor (HIF)-1α transcriptional activity assay. Cells were untreated or treated with bicalutamide (Bic) at doses of 30 and 60 μM for 3–24 h and *hif-1α* transcriptional activity was analyzed with a commercial kit (Cayman). Hif-1α transcriptional activity had only increased at 24 h after treatment with Bic. (**b**) Quantitative polymerase chain reaction (qPCR) for *hif-1α* mRNA. Rat mesangial cells (RMCs) were treated with Bic at doses of 30 or 60 μM for 24 h. Total RNA was extracted and reverse-transcribed to cDNA. *hif-1α* mRNA was detected according the protocol of a commercial SYBR green QPCR kit (Taigen Bioscience). *Hif-1α* mRNA dose-dependently increased. (**c**) Western blot analysis of the HIF-1α protein in RMC cells. Cells were treated with the indicated doses of Bic for 0–48 h. Nuclear proteins were subfractionated from total cell lysates and loaded onto SDS-PAGE gels. Blots were detected using antibodies of HIF-1α and lamin B. Lamin B was used as an internal control. Levels of HIF-1α protein expression were quantified by Image J software and normalized to that of lamin B. Representative images are shown and quantitative data are from three replicates. Data are expressed as the mean±standard deviation. * *p* < 0.05, ** *p* < 0.01 compared to untreated cells; ^#^ *p* < 0.05, ^##^ *p* < 0.01 compared to 30 μM Bic-treated cells.

The HIF-1α protein is a critical transcription factor in adaptive responses to hypoxia. Under hypoxia, stabilized HIF-1α is translocated into nuclei, where it heterodimerizes with HIF-1β, is then activated and accumulates in cells [29]. Compared to the control (0 μM Bic), *hif-1α* transcriptional activity was found to have increased only at 24 h after treatment with Bic and an obvious difference was also found between treatment with 30 and 60 μM Bic (Figure 5a). Simultaneously, the quantity of *hif-1α* mRNA at 24 h was found to have increased in a dose-dependent manner (Figure 5b) and triggering of *hif-1α* mRNA seemed to occur simultaneously with the increase in *hif-1α* transcription activity (Figure 5a, b). Coincidently, amounts of HIF-1α were also elevated to 1.26- and 1.41-fold at 24 h by 30 and 60 μM Bic, respectively. However, HIF-1α induced by Bic rapidly decreased to 0.83- and 0.4-fold at 48 h after treatment (*p* < 0.05) (Figure 5c).

Much of the literature mentions some solid supporting evidence for this finding. In response to acute hypoxia, HIF-1α rapidly accumulates in cells due to inactivation of oxygen-sensitive prolyl hydroxylase [76]. Prolonged hypoxia initiates CHIP (a prolyl hydroxylase-independent E3 ligase)-mediated HIF-1α degradation and lowers its cellular levels [77,78]. HIF-1α and its downstream targets may offer only short-term protection following ischemia-reperfusion injury in myocardial cells [79]. On the other hand, HIF-1α also triggers apoptosis, possibly in cases where cellular responses are insufficient to meet energy demands under hypoxic conditions [80].

### 2.6. Bic Induced Cell Apoptosis via BNIP3-Caspase 3 Signaling and Modulation of HIF-1α Partially Inhibited Cell Death in RMCs

**Figure 6:**
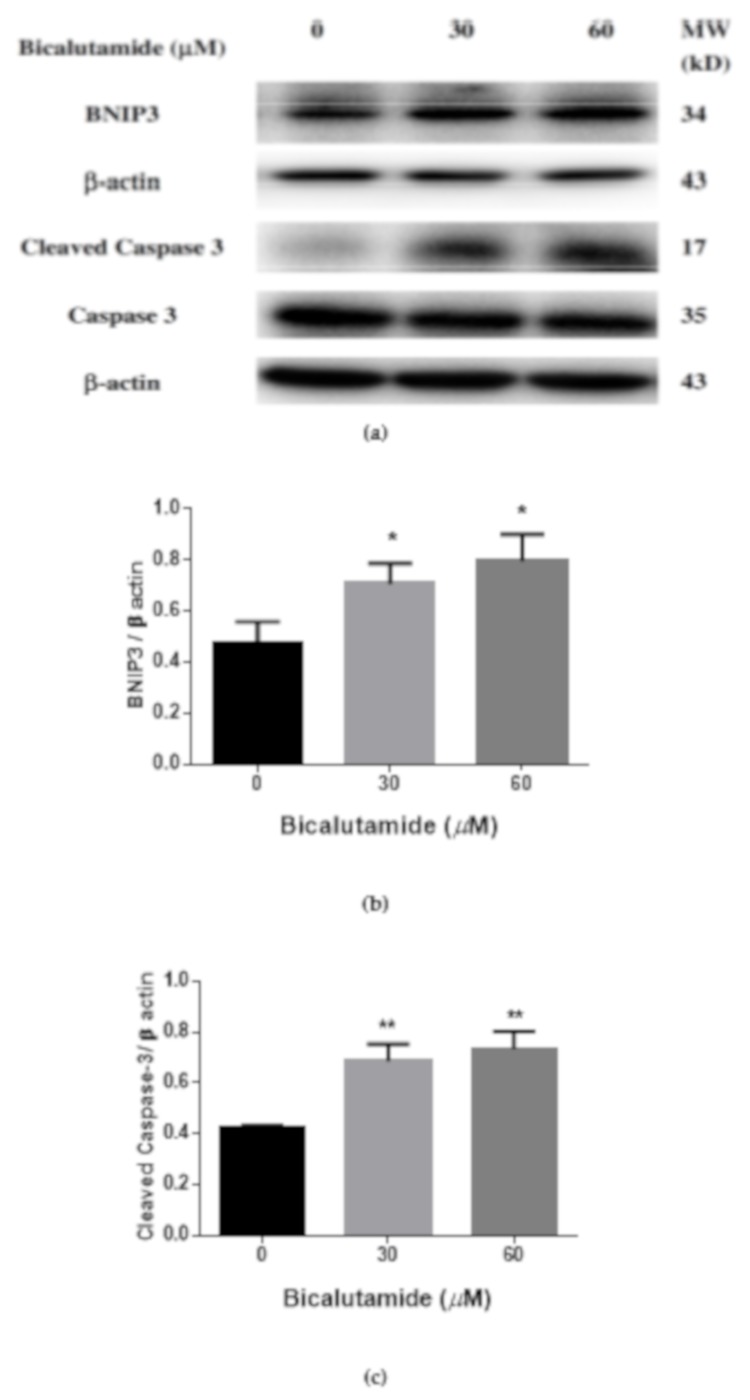

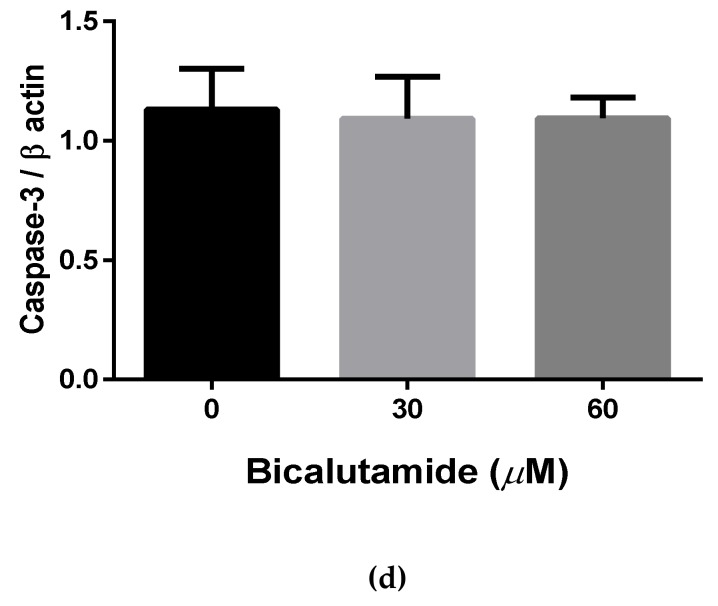
Expression and the quantitation of BCL2/adenovirus E1B 19 kDa protein-interacting protein 3 (BNIP3), cleaved-caspase-3 and caspase-3 in rat mesangial cells (RMCs) after being treated with bicalutamide (Bic) at 30 and 60 μM for 24 h. Total RMC lysates were loaded onto Western blots for analysis of BNIP3, caspase-3, cleaved caspase-3 and β-actin. β-actin served as the internal control. (**a**) Western blot of BNIP3 and cleaved caspase-3. (**b**) Bar diagram of BNIP3. (**c**) Bar diagram of cleaved caspase-3. (**d**) Bar diagram of caspase-3. Cleaved caspase 3 was significantly upregulated at 24 h after treatment. In contrast, caspase-3 was unaffectd (**d**). Data were obtained from triplicate determinations and results are expressed as the mean ± standard deviation. * *p* < 0.05, ** *p* < 0.01 compared to the control.

HIF target genes are involved in cellular apoptosis and profibrotic mechanisms [81]. BNIP3, which belongs to the Bcl-2 protein family of proapoptotic proteins that regulate programmed cell death, is expressed in nuclei and the cytoplasm during hypoxia, acting as a hypoxia-induced protein involved in cell death and survival [82]. BNIP3 expression by mitochondria induces apoptosis and can overcome cell death suppressed by Bcl-2 [82]. As shown in Figure 6a, b, the BNIP-3 signal was respectively upregulated by Bic at 30 and 60 μM by 1.48- and 1.67-fold (*p* < 0.05). In parallel, Bic was found to upregulate cleaved-caspase-3 at 30 and 60 μM (Figure 6a, c, d), evidencing an increased apoptotic effect by Bic.

**Figure 7:**
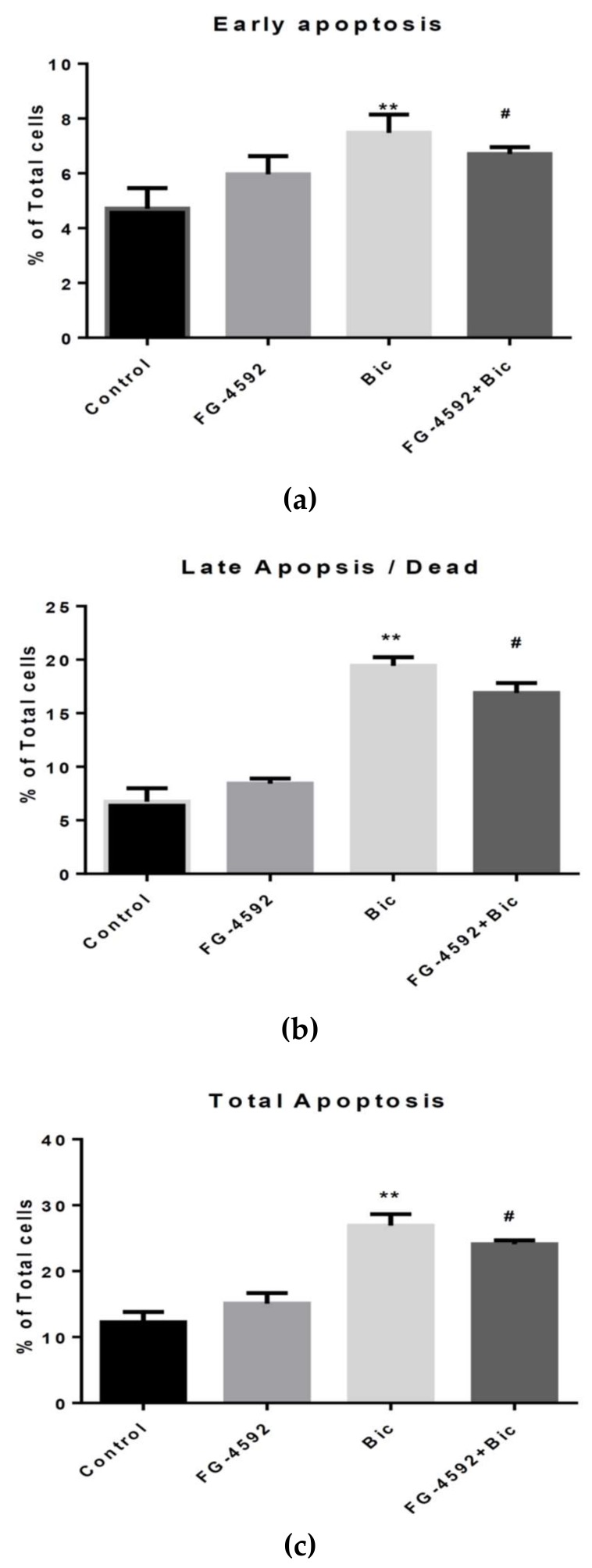
Annexin V analysis of apoptosis of rat mesangial cells (RMCs) mediated by bicalutamide (Bic) and FG4592. Cells were co-treated with Bic (60 μM) for 24 h in the presence and absence of FG4592 (2.5 μM). (**a**) Upper panel: early apoptosis. (**b**) Middle panel: late apoptosis/dead. (**c**) Lower panel: total apoptosis. A statistical analysis of the cytometric data showed that FG4592 partially reversed apoptotic cells induced by Bic. Triplicate data were statistically analyzed and results are expressed as the mean ± standard deviation. ** *p* < 0.01 compared to the control. ^#^ *p* < 0.05 compared to 60 μM Bic-treated cells.

Further, we used Roxadustat (FG-4592), an inhibitor of prolyl hydroxylase, to examine whether this compound could abolish the apoptotic effect of HIF-1α. Results showed that Bic caused 1.59, 2.88 and 2.20-fold increase of early, late and total apoptosis compared to control respectively (Figure 7). The significant improvements of RMCs co-treated with Bic and FG-4592 are shown by 1.42, 2.51 and 1.97-fold of cells in the early, late and total apoptosis compared to control respectively (Figure 7). The FG-4592 was only partially effective in reducing apoptosis mediated by Bic, implying that multiple mechanisms excluding HIF-1α are involved in the apoptotic phenomenon. Emerging evidence has suggested that FG-4592 plays the role in treating CKD anemia [83], the co-treatment of FG-4592 might protect the RMC cells against Bic-induced kidney injury.

**Figure 8:**
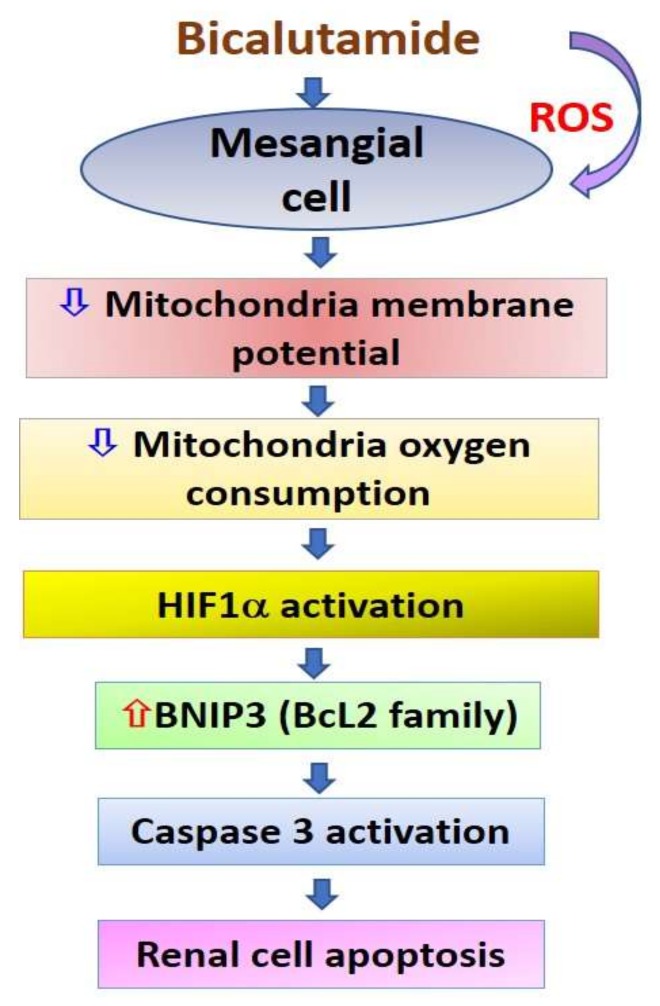
A graphic summary of bicalutamide-induced renal mesangial cell damage via mitochondrial dysfunction.

In summary, Bic exhibited a certain degree of cytotoxicity even at regular doses prescribed for patients. Bic caused LDH release, upregulation of KIM and downregulation of N-cadherin. Bic elicited excessive production of ROS, membrane potential depolarization, mitochondrial swelling and a decreased mitochondrial population and consequently, the OCR and ATP production were dose-dependently inhibited. In parallel, Bic dose- and time-dependently upregulated *hif*-1α transcription and *hif-1α* mRNA. A summary of Bic-induced renal mesangial cell damage is shown in Figure 8. However, the appearance of HIF-1α reached peak values at 24 h, then rapidly declined to 48 h. On the other hand, Bic dose-dependently upregulated BNIP3, cleaved caspase-3 and elicited cell apoptosis, including early and late apoptosis. Furthermore, apoptosis induced by Bic was partially ameliorated by FG-4592.

Meanwhile, to prevent adverse effects induced by Bic, some nutraceutic compounds are suggested to be applied as adjuvant therapy to ameliorate or prevent the mitochondrial dysfunction, such as coenzyme Q, which is also dependent on vitamins B2, B6, B12 and C, folic acid, pantothenic acid and niacinamide [84]. As is well known, vitamins B1, B2 and B6, niacin, biotin, folic acid and pantothenic acid are all important modulators or cofactors in metabolic pathways that assist mitochondrial respiration and energy production. In addition, vitamins C and E, niacin and folic acid are effective scavengers of free radicals and diminish the formation of mitochondrial oxidants and the aging of mitochondria.

## 3. Materials and Methods

### 3.1. Chemicals

Bicalutamide (Bic), 3-(4,5-dimethylthiazol-2-yl)-2,5-diphenyltetrazolium bromide (MTT), Dulbecco’s modified Eagle medium (DMEM), tetramethyl ethylene diamine (TEMED) and FG4592 were provided by Sigma-Aldrich (St. Louis, MO, USA). The enhanced chemiluminescence (ECL) system was a product of Merck Millipore (Billerica, MA, USA). The PRO-PREP Protein Extraction Solution was provided by iNtRON Biotech. (Kyungki-Do, Korea). JC-1, dihydroethidium (DHE) and dichlorodihydrofluorescein diacetate (DCFDA) were provided by Millipore Sigma (St. Louis, MO, USA). The 2% charcoal-containing fetal bovine serum (FBS) was provided by GeneTex (Irvine, CA, USA).

### 3.2. Cell Culture

A rat mesangial cell (RMC) line was provided by the Bioresource Collection and Research Center (Hsinchu, Taiwan). RMCs were cultured in modified DMEM containing 25 mM glucose, 15% FBS, 4 mM L-glutamine, 1.5 g/L of sodium bicarbonate, 0.4 mg/mL of G418 and 0.4% phosphate-buffered saline (PBS) and incubated at 37 °C under a 5% CO_2_ atmosphere. After cells had completely adhered, the medium was replaced with 2% charcoal FBS DMEM and incubated overnight (for 12–16 h). Then the indicated drugs were applied at 30 and 60 μM Bic that was previously prepared in DMEM with 2% charcoal FBS. The medium was replaced with fresh medium and cells were incubated for different periods as indicated.

### 3.3. Lactic Dehydrogenase (LDH) Assay

Cells were cultured as described above. Drugs were applied and the LDH assay was carried out after 48 h using a Cayman LDH Cytotoxicity Assay Kit (cat. No. K601170). The optical density was measured at 490 nm.

### 3.4. Flowcytometric Analysis for DHE-Oxidative Stress

Following a method described by Owusu-Ansah et al. [85], a DHE oxidative stress assay was carried out. As a cell-permeable reagent, DHE is a red dye that is useful for detecting ROS. All cell groups were cultured as indicated. Bic at the indicated doses was incubated for 60 min. The medium was removed and cells were harvested with the addition of tyrosine. The fluorescent intensity of DHE was monitored under an excitation wavelength of 480–520 nm and an emission wavelength of 570–600 nm using the MCH100111 Mouse^™^ Oxidative Stress Kit (Merck-Millipore, Dresden, Germany).

### 3.5. Fluorescent Staining for DCFCA-Oxidative Stress

According to Owusu-Ansah et al. [85] and following instructions of the manufacturer (Merck-Millipore), cells were cultured as described above. Bic at the indicated doses was applied. Media in samples obtained at 10 and 60 min were removed and replaced with 200 μL of DMEM containing 20 μM DCFDA and 2% FBS. Cells were incubated at 37 °C under a 5% CO_2_ atmosphere for 1 h. After removing the medium, cells were rinsed with PBS several times and cells were observed under a fluorescent microscope (Olympus, Tokyo, Japan).

### 3.6. JC-1 Assay for the Mitochondrial Membrane Potential (MMP)

RMCs were trypsinized and seeded onto 3.5-cm culture dishes. When cells had completely adhered, the medium was replaced with fresh 2% charcoal FBS DMEM and incubated overnight. Bic was added at the indicated concentrations and incubated for 24 h. The medium was then removed and replaced with 2% charcoal FBS DMEM containing 1 μg/mL of JC-1. Cells were incubated at 37 °C for 30 min under a 5% CO_2_ atmosphere. The medium was removed and observed with a Leica TCS SP5 Confocal Spectral Microscope Imaging System (Buffalo Grove, IL, USA). JC-1 green-fluorescent monomers that formed at depolarized membrane potentials were detected at Ex/Em = 514/529 nm and orange-fluorescent J-aggregates that formed at hyperpolarized membrane potentials were detected at Ex/Em = 585/590 nm.

Alternately, FCCP (10 μM), a protonophore, was added to untreated cell cultures at a concentration of 50 nM to depolarize mitochondrial membranes as the positive control. Approximately 10 min after the addition of the uncoupler, cells were illuminated at Ex/Em (nm) = 514/529 nm for green monomers and Ex/Em (nm) = 585/590 nm for J-aggregates.

### 3.7. Nanolive 3D Cell Explorer

RMCs (400 µl) were seeded into 35-mm tissue culture dishes at a density of 6 × 10^4^ cells/dish overnight. After cells had totally adhered, the medium was replaced with 2% charcoal FBS-DMEM and incubated overnight. Then RMCs were treated with 60 μM Bic for 48 h or left untreated, after which cells were transferred to phenol red-free medium. Dishes were then placed on a Nanolive 3D cell explorer (NanoLive, Lausanne, Switzerland) and images are taken at 600 × magnification.

### 3.8. Transmission Electron Microscopy (TEM)

RMCs (3 × 10^5^) were seeded into poly-L-lysine-coated two-well chamber slides and treated with 30 and 60 μM Bic for 24–48 h. Cells were fixed in 0.1 M cacodylate solution containing 2% paraformaldehyde and 2.5% glutaraldehyde for 1 h. After removing the fixative solution, cells were rinsed thrice with buffer solution containing 0.1 M cacodylate and 7% sucrose, for 15 min each time. Specimens were stained with 1% OsO_4_ contrast solution (in 0.1 M cacodylate buffer) for 1–2 h and then subjected to a gradient ethanol dehydration method (i.e., 70% ethanol for 15 min; 80% ethanol for 15 min; 90% ethanol for 15 min; 95% ethanol twice, each time for 15 min; and finally 100% ethanol thrice, each time for 15 min). Dehydrated specimens were treated thrice with propylene oxide (PO), each time for 10 min. The resin was evacuated under a vacuum for 8 h and PO/resin (1:1) was mixed and evacuated under a vacuum overnight. Specimens were embedded and heated to 62 °C in the oven for 48–72 h until completely set, then reshaped, sliced and analyzed by HT7700 TEM (Hitachi, Tokyo, Japan) for access to intracellular ultrastructures.

### 3.9. Cellular Bioenergetic Analysis using the Seahorse XF Analyzer

The entire protocol was carried out following instructions of the manufacturer (Agilent Technology, Santa Clara, CA, USA). RMCs were seeded onto a collagen-coated XF 96-well cell culture microplate (2.5 × 10^4^ cells/100 μL of medium/well) and incubated overnight (at 37 °C in 5% CO_2_). Bic at indicated doses was applied for 24 h. Then, cell culture medium was replaced with 175 μL of unbuffered Seahorse XF Base medium supplemented with 25 mM of glucose, 2 mM of L-glutamine and 1 mM of sodium pyruvate at 1 h before the measurement. Prior to the measurement, the Seahorse XF analyzer gently mixed the assay medium in each well for 10 min to enable the oxygen partial pressure to reach equilibrium. The OCR was then measured three times to establish a baseline rate prior to an acute injection of Bic. The following was the procedure for the Cell Mito Stress Test (Agilent Technology): (1) basal respiration was measured in unbuffered medium; (2) oligomycin (1 μM), an inhibitor of ATP synthesis, was injected to determine respiration linked to ATP production; (3) FCCP (2 μM), a mitochondrial oxidative phosphorylation uncoupler, was added to measure maximal respiration; and (4) antimycin A and rotenone (1 μM each) were applied in combination to block respiration due to the simultaneous inhibition of complexes III and I, respectively [86]. The OCR and basal extracellular acidification rate (ECAR) were measured in real time. The basal respiration, proton leakage, ATP-linked OCR, maximal respiration and spare respiratory capacity were calculated. All assays used DMSO at ≤0.5% as a vehicle control [69].

### 3.10. Measurement of HIF-1α Transcriptional Activity

HIF-1α transcriptional activity was analyzed by an HIF-1α transcription factor assay kit (Cayman, Ann Arbor, MI, USA). RMCs (4 × 10^6^ cells/mL) were seeded and grown in 10-cm culture dishes overnight. Then Bic at 30 or 60 μM was applied for 3, 6 and 24 h. Transcription activity of HIF in the nuclear extract was detected at OD 450 nm.

### 3.11. Quantitative Polymerase Chain Reaction (qPCR) for hif-1α mRNA

RMCs were cultured and treated with Bic as indicated. Total RNA was extracted, isolated and purified with a Novel Gene Total RNA Purification Mini Kit (Yu-Shing Biotech, Taipei, Taiwan) at 6 and 24 h. After the amount of RNA was quantified using a Nano-Drop 1000 Spectrophotometer (ThermoFisher Scientific, Waltham, MA, USA), 1 μg of RNA was subjected to a qPCR to produce complementary (c)DNA (GoScript™ Reverse Transcription System, Promega, Madison, WI, USA). A real-time thermocycler (Analytik Jena, Upland, CA, USA) was used to perform the reactions as follows—1 cycle of pre-denaturation at 95 °C for 3 min and then 35 cycles sequentially processed according to the sequence: denaturation at 95 °C for 15 s; annealing at 59 °C for 30 s; and extension at 72 °C for 30 s, followed by one cycle final extension at 72 °C for 10 min and one cycle cool-down at 4 °C for 10 min. The LabStarTM SYBR QPCR Kit (Taigen Bioscience, Taipei, Taiwan) was adopted for fluorescence detection. The respective primers used were: *hif1α*, forward 5’-CAATTCTCCAAGCCCTCCGA-3’ and reverse 5’-ATTCATCAGTGGTGGCAGTTG-3’ and *β-actin*, forward 5’-ATGGTGGGTATGGGTCAGAA-3’ and reverse 5’-CACACGCAGCTCATTGTAGA-3’. Amplifications was normalized to *β-actin* using the 2^−^^△△^CT method [87].

### 3.12. Western Blotting

RMCs were treated with Bic for 24–48 h as indicated. Extraction of cytoplasmic and nuclear proteins was carried out as instructed by the cytoplasmic and nuclear protein extraction kit (Biotools, New Taipei City, Taiwan). The proteins obtained were transferred into new Eppendorf flasks and stored at −80 °C. A previously described standard protocol was used [88]. Specific primary antibodies (1:1000× with 0.1% TBST) used in this study included N-cadherin (ab76011) from Abcam (Cambridge, MA, USA); Lamin-B1 (ab133741), HIF-1α (GTX127309), BNIP-3 (GTX111876) from GeneTex (Irvine, CA, USA); KIM-1 (ab190696) from Abcam (Cambridge, UK); Caspase-3 (nos. 9661 and 9662) a product of Cell Signaling Technology (Danvers, MA, USA); and β-actin (AC-15) and GAPDH (NB300-221) purchased from Novus (Abingdon, UK). The Western blot analysis was repeated at least three times. Original Western blot images are provided in Supplemental Figures S2–S5.

### 3.13. Annexin V assay for Apoptosis

In order to elucidate the role of HIF-1 in cell apoptosis, FG-4592 (Sigma-Aldrich, St. Louis, MO, USA) was used to interfere with the effect of HIF-1. FG-4592 is a small molecule that acts as a modulator of polyhydroxylase which can degrade HIF [83].

The cell apoptosis analysis was carried out with an MCH100105 Muse^®^ Annexin V and Dead Cell Kit on a Muse Cell Analyzer (Merck-Millipore, Dresden, Germany) following the protocols of the manufacturer. In brief, RMCs were seeded onto six-well plates at a density of 4 × 10^5^ cells/well in 2% charcoal FBS. The medium was aspirated off, replaced with new medium containing the target medicine Bic (at 0 and 60 μM, respectively) in 2% charcoal FBS and co-incubated with FG-4592 (at 2.5 μM) for 24 h. Cells with medium were harvested, treated with trypsin for 3 min, transferred to a centrifuge tube and centrifuged at 1000× *g* for 5 min. The supernatant was aspirated off. A PBS wash was added, agitated well and centrifuged for 5 min at 1000× *g*. The supernatant was aspirated off and new medium containing 2% charcoal FBS (1 mL) was added and mixed well. To an aliquot of 100 μL of the culture, 100 μL of the Annexin V solution (Millipore MCH100105) was added and mixed well. The mixture was left to react for 20 min in the dark avoiding direct sunlight. The solution was subjected to Muse and analyzed with Muse 1.3.1. software (Luminex, Chicago, IL, USA).

### 3.14. Statistical Analysis

All data were analyzed by Student’s *t*-test in SPSS 10.0 software (SPSS, Chicago, IL, USA). An analysis of variance (ANOVA) was also adopted with Tukey’s test to analyze variances and significances of difference between paired means. The significance level was judged by the *p* value. * *p* < 0.05, ** *p* < 0.01, *** *p* < 0.005.

## 4. Conclusions

Bic has been used as one of the effective antiandrogens. The regular dose is around 50–250 mg/day. However, even at such doses, it has frequently been reported to have adverse effects associated with cardiovascular and renal damage. We showed that Bic selectively damaged RMC kidney cells, the etiology of which was due to the immense production of ROS and the mitochondrial damage that resulted in a decreased OCR. Speculatively, patients afflicted with renal diseases and DM may be more severely injured than those without those complications. Moreover, prolonged Bic treatment may induce upregulation of HIF-1α, KIM-1 and BNIP3, resulting in cell apoptosis in the renal organ. Consequently, when Bic is prescribed for patients, it is warranted to take these adverse effects of Bic into consideration, in particular, for DM patients.

## Supplementary Materials

The following are available online at https://www.mdpi.com/1422-0067/21/9/3400/s1. Figure S1. Cluster plot of the pathological score of in vivo experimental rats with diabetic nephropathy; Original Western blot images are provided in Supplemental Figures S2–S5.

## Abbreviations

ADT	androgen deprivation therapy
AKI	acute kidney injury
Bic	bicalutamide
BNIP3	Bcl-2 interacting protein 3
CAB	combined androgen blockade
CVD	cardiovascular diseases
DCFDA	2’,7’-dichlorofluorescin diacetate
DHE	dihydroethidium
DM	diabetes mellitus
DMEM	Dulbecco’s modified Eagle medium
ECAR	extracellular acidification rate
ECL	enhanced chemiluminescence
HIF-1α	hypoxia-inducible factor 1-alpha
KIM-1	kidney injury molecule-1
LDH	lactic dehydrogenase
MMP	mitochondrial membrane potential
MTT	3-(4,5-dimethylthiazol-2-yl)-2,5-diphenyltetrazolium bromide
OCR	oxygen consumption rate
OXPHOS	oxidative phosphorylation
PCa	prostate cancer
RMC	rat mesangial cell
ROS	reactive oxygen species
TEM	transmission electron microscopy

## References

[1] Bracci U., Di Silverio F., Martini L., Motta M. (1977). Androgens and Antiandrogens. Role of Cyproterone Acetate in Urology.

[2] Akaza H. (1999). A new anti-androgen, bicalutamide (Casodex), for the treatment of prostate cancer—Basic clinical aspects. Gan Kagaku Ryoho.

[3] Floyd M.S., Teahan S.J., Fitzpatrick J.M., Watson R.W. (2009). Differential mechanisms of bicalutamide-induced apoptosis in prostate cell lines. Prostate Cancer Prostatic Dis..

[4] Klotz L., Drachenberg D., Singal R., Aprikian A., Fradet Y., Kebabdjian M., Zarenda M., Chin J. (2014). An open-label, phase 2 trial of bicalutamide dose escalation from 50 mg to 150 mg in men with CAB and castration resistance. A Canadian Urology Research Consortium Study. Prostate Cancer Prostatic Dis..

[5] Chitturi S., Farrell G.C. (2013). Adverse Effects of Hormones and Hormone Antagonists on the Liver. Drug-Induced Liver Disease.

[6] Saito S. (2020). Successful recovery from multiple organ failure associated with bicalutamide and leuprorelin acetate for prostate cancer. Urol. Case Rep..

[7] Antiandrogens (2012). LiverTox: Clinical and Research Information on Drug-Induced Liver Injury.

[8] Yun G.Y., Kim S.H., Kim S.W., Joo J.S., Kim J.S., Lee E.S., Lee B.S., Kang S.H., Moon H.S., Sung J.K. (2016). Atypical onset of bicalutamide-induced liver injury. World J. Gastroenterol..

[9] Bosco C., Bosnyak Z., Malmberg A., Adolfsson J., Keating N.L., Van Hemelrijck M. (2015). Quantifying observational evidence for risk of fatal and nonfatal cardiovascular disease following androgen deprivation therapy for prostate cancer: A meta-analysis. Eur. Urol..

[10] Chatelain C., Rousseau V., Cosaert J. (1994). French multicentre trial comparing Casodex (ICI 176,334) monotherapy with castration plus nilutamide in metastatic prostate cancer: A preliminary report. Eur. Urol..

[11] Schellhammer P., Sharifi R., Block N., Soloway M., Venner P., Patterson A.L., Sarosdy M., Vogelzang N., Jones J., Kolvenbag G. (1995). A controlled trial of bicalutamide versus flutamide, each in combination with luteinizing hormone-releasing hormone analogue therapy, in patients with advanced prostate cancer. Casodex Combination Study Group. Urology.

[12] Kennealey G.T., Furr B.J. (1991). Use of the nonsteroidal anti-androgen Casodex in advanced prostatic carcinoma. Urol. Clin. North Am..

[13] eHealthMe.com (2019). Casodex and Kidney Failure—From FDA Reports. https://www.ehealthme.com/ds/casodex/kidney-failure/.

[14] Peng C.C., Chen C.Y., Chen C.R., Chen C.J., Shen K.H., Chen K.C., Peng R.Y. (2019). Renal Damaging Effect Elicited by Bicalutamide Therapy Uncovered Multiple Action Mechanisms As Evidenced by the Cell Model. Sci. Rep..

[15] (2012). LiverTox: Clinical and Research Information on Drug-Induced Liver Injury. Bicalutamide. https://www.ncbi.nlm.nih.gov/books/NBK547970/.

[16] Ikeyama Y., Sato T., Takemura A., Sekine S., Ito K. (2020). Successful energy shift from glycolysis to mitochondrial oxidative phosphorylation in freshly isolated hepatocytes from humanized mice liver. Toxicol. In Vitro.

[17] Kashimshetty R., Desai V.G., Kale V.M., Lee T., Moland C.L., Branham W.S., New L.S., Chan E.C., Younis H., Boelsterli U.A. (2009). Underlying mitochondrial dysfunction triggers flutamide-induced oxidative liver injury in a mouse model of idiosyncratic drug toxicity. Toxicol. Appl. Pharmacol..

[18] Basaria S. (2008). Androgen deprivation therapy, insulin resistance, and cardiovascular mortality: An inconvenient truth. J. Androl..

[19] Keating N.L., O’Malley A.J., Smith M.R. (2006). Diabetes and cardiovascular disease during androgen deprivation therapy for prostate cancer. J. Clin. Oncol..

[20] Smith M.R., Lee H., Nathan D.M. (2006). Insulin sensitivity during combined androgen blockade for prostate cancer. J. Clin. Endocrinol. Metab..

[21] Karalliedde J., Gnudi L. (2016). Diabetes mellitus, a complex and heterogeneous disease, and the role of insulin resistance as a determinant of diabetic kidney disease. Nephrol. Dial. Transplant..

[22] Sasson A.N., Cherney D.Z. (2012). Renal hyperfiltration related to diabetes mellitus and obesity in human disease. World J. Diabetes.

[23] Mogensen C.E. (1986). Early glomerular hyperfiltration in insulin-dependent diabetics and late nephropathy. Scand. J. Clin. Lab. Invest..

[24] Mogensen C.E. (1971). Glomerular filtration rate and renal plasma flow in short-term and long-term juvenile diabetes mellitus. Scand. J. Clin. Lab. Invest..

[25] Lurbe E., Redon J., Kesani A., Pascual J.M., Tacons J., Alvarez V., Batlle D. (2002). Increase in nocturnal blood pressure and progression to microalbuminuria in type 1 diabetes. N. Engl. J. Med..

[26] Gnudi L., Thomas S.M., Viberti G. (2007). Mechanical forces in diabetic kidney disease: A trigger for impaired glucose metabolism. J. Am. Soc. Nephrol..

[27] Yu S.M., Bonventre J.V. (2018). Acute Kidney Injury and Progression of Diabetic Kidney Disease. Adv. Chronic Kidney Dis..

[28] Lekas E., Bergh A., Damber J.E. (2000). Effects of finasteride and bicalutamide on prostatic blood flow in the rat. BJU Int..

[29] Shu S., Wang Y., Zheng M., Liu Z., Cai J., Tang C., Dong Z. (2019). Hypoxia and Hypoxia-Inducible Factors in Kidney Injury and Repair. Cells.

[30] Song Y.R., You S.J., Lee Y.M., Chin H.J., Chae D.W., Oh Y.K., Joo K.W., Han J.S., Na K.Y. (2010). Activation of hypoxia-inducible factor attenuates renal injury in rat remnant kidney. Nephrol. Dial. Transplant..

[31] Fine L.G., Bandyopadhay D., Norman J.T. (2000). Is there a common mechanism for the progression of different types of renal diseases other than proteinuria? Towards the unifying theme of chronic hypoxia. Kidney Int. Suppl..

[32] Nangaku M. (2006). Chronic hypoxia and tubulointerstitial injury: A final common pathway to end-stage renal failure. J. Am. Soc. Nephrol..

[33] Tanaka T., Miyata T., Inagi R., Kurokawa K., Adler S., Fujita T., Nangaku M. (2003). Hypoxia-induced apoptosis in cultured glomerular endothelial cells: Involvement of mitochondrial pathways. Kidney Int..

[34] Okamoto A., Sumi C., Tanaka H., Kusunoki M., Iwai T., Nishi K., Matsuo Y., Harada H., Takenaga K., Bono H. (2017). HIF-1-mediated suppression of mitochondria electron transport chain function confers resistance to lidocaine-induced cell death. Sci. Rep..

[35] Fu Q., Colgan S.P., Shelley C.S. (2016). Hypoxia: The Force that Drives Chronic Kidney Disease. Clin. Med. Res..

[36] Tanaka T. (2017). A mechanistic link between renal ischemia and fibrosis. Med. Mol. Morphol..

[37] Rothermund C.A., Gopalakrishnan V.K., Eudy J.D., Vishwanatha J.K. (2005). Casodex treatment induces hypoxia-related gene expression in the LNCaP prostate cancer progression model. BMC Urol..

[38] Wei P.Z., Szeto C.C. (2019). Mitochondrial dysfunction in diabetic kidney disease. Clin. Chim. Acta.

[39] McBride H.M., Neuspiel M., Wasiak S. (2006). Mitochondria: More than just a powerhouse. Curr. Biol..

[40] Burd J.F., Usategui-Gomez M. (1973). A colorimetric assay for serum lactate dehydrogenase. Clin. Chim. Acta.

[41] Kang S.K., Ha C.Y., Cho K.H., Park S.K., Kim U.H. (1991). Changes of lactate dehydrogenase and its isoenzyme activity in renal diseases. Nephron.

[42] Ichimura T., Hung C.C., Yang S.A., Stevens J.L., Bonventre J.V. (2004). Kidney injury molecule-1: A tissue and urinary biomarker for nephrotoxicant-induced renal injury. Am. J. Physiol. Renal. Physiol..

[43] Han W.K., Bailly V., Abichandani R., Thadhani R., Bonventre J.V. (2002). Kidney Injury Molecule-1 (KIM-1): A novel biomarker for human renal proximal tubule injury. Kidney Int..

[44] Zhao X., Zhang Y., Li L., Mann D., Imig J.D., Emmett N., Gibbons G., Jin L.M. (2011). Glomerular expression of kidney injury molecule-1 and podocytopenia in diabetic glomerulopathy. Am. J. Nephrol..

[45] Nameta M., Yaoita E., Kato N., Zhao L., Zhang Y., Fujinaka H., Xu B., Yoshida Y., Yamamoto T. (2009). Mesangial cells connected by the N-cadherin-catenin system in the rat kidney. Nephron Exp. Nephrol..

[46] Whaley-Connell A., Habibi J., Panfili Z., Hayden M.R., Bagree S., Nistala R., Hyder S., Krueger B., Demarco V., Pulakat L. (2011). Angiotensin II activation of mTOR results in tubulointerstitial fibrosis through loss of N-cadherin. Am. J. Nephrol..

[47] Blindt R., Bosserhoff A.K., Dammers J., Krott N., Demircan L., Hoffmann R., Hanrath P., Weber C., Vogt F. (2004). Downregulation of N-cadherin in the neointima stimulates migration of smooth muscle cells by RhoA deactivation. Cardiovasc. Res..

[48] Lee E.C., Zhan P., Schallhom R., Packman K., Tenniswood M. (2003). Antiandrogen-induced cell death in LNCaP human prostate cancer cells. Cell Death Differ..

[49] Dekkers C.C.J., Petrykiv S., Laverman G.D., Cherney D.Z., Gansevoort R.T., Heerspink H.J.L. (2018). Effects of the SGLT-2 inhibitor dapagliflozin on glomerular and tubular injury markers. Diabetes Obes. Metab..

[50] Waikar S.S., Murray P., Singh A.K. (2018). Core Concepts in Acute Kidney Injury.

[51] Pawar R.D., Pitashny M., Gindea S., Tieng A.T., Levine B., Goilav B., Campbell S.R., Xia Y., Qing X., Thomas D.B. (2012). Neutrophil gelatinase-associated lipocalin is instrumental in the pathogenesis of antibody-mediated nephritis in mice. Arthritis Rheum..

[52] St-Pierre J., Buckingham J.A., Roebuck S.J., Brand M.D. (2002). Topology of superoxide production from different sites in the mitochondrial electron transport chain. J. Biol. Chem..

[53] Chance B., Sies H., Boveris A. (1979). Hydroperoxide metabolism in mammalian organs. Physiol. Rev..

[54] Wardman P. (2007). Fluorescent and luminescent probes for measurement of oxidative and nitrosative species in cells and tissues: Progress, pitfalls, and prospects. Free Radic. Biol. Med..

[55] Gomes A., Fernandes E., Lima J.L. (2005). Fluorescence probes used for detection of reactive oxygen species. J. Biochem. Biophys. Methods.

[56] Nunes J.J., Pandey S.K., Yadav A., Goel S., Ateeq B. (2017). Targeting NF-kappa B Signaling by Artesunate Restores Sensitivity of Castrate-Resistant Prostate Cancer Cells to Antiandrogens. Neoplasia.

[57] Perry S.W., Norman J.P., Barbieri J., Brown E.B., Gelbard H.A. (2011). Mitochondrial membrane potential probes and the proton gradient: A practical usage guide. Biotechniques.

[58] Arya A., Khandelwal K., Ahmad H., Laxman T.S., Sharma K., Mittapelly N., Agrawal S., Bhatta R.S., Dwivedi A.K. (2016). Co-delivery of hesperetin enhanced bicalutamide induced apoptosis by exploiting mitochondrial membrane potential via polymeric nanoparticles in a PC-3 cell line. RSC Adv..

[59] Dispersyn G., Nuydens R., Connors R., Borgers M., Geerts H. (1999). Bcl-2 protects against FCCP-induced apoptosis and mitochondrial membrane potential depolarization in PC12 cells. Biochim. Biophys. Acta.

[60] Fosslien E. (2001). Mitochondrial medicine--molecular pathology of defective oxidative phosphorylation. Ann. Clin. Lab. Sci..

[61] Zhao H., Liu Y.J., Liu Z.R., Tang D.D., Chen X.W., Chen Y.H., Zhou R.N., Chen S.Q., Niu H.X. (2017). Role of mitochondrial dysfunction in renal fibrosis promoted by hypochlorite-modified albumin in a remnant kidney model and protective effects of antioxidant peptide SS-31. Eur. J. Pharmacol..

[62] Dominy J.E., Puigserver P. (2013). Mitochondrial biogenesis through activation of nuclear signaling proteins. Cold Spring Harb. Perspect. Biol..

[63] Yoo S.M., Jung Y.K. (2018). A Molecular Approach to Mitophagy and Mitochondrial Dynamics. Mol. Cells.

[64] Haga H.J., Andersen K.J., Rygh T., Iversen B.M., Matre R. (1988). Changes in lysosome populations in the rat kidney cortex induced by experimental proteinuria. Int. J. Biochem..

[65] Widlansky M.E., Wang J., Shenouda S.M., Hagen T.M., Smith A.R., Kizhakekuttu T.J., Kluge M.A., Weihrauch D., Gutterman D.D., Vita J.A. (2010). Altered mitochondrial membrane potential, mass, and morphology in the mononuclear cells of humans with type 2 diabetes. Transl. Res..

[66] Che R., Yuan Y., Huang S., Zhang A. (2014). Mitochondrial dysfunction in the pathophysiology of renal diseases. Am. J. Physiol. Ren. Physiol..

[67] Cogliati S., Enriquez J.A., Scorrano L. (2016). Mitochondrial Cristae: Where Beauty Meets Functionality. Trends Biochem. Sci..

[68] Wakabayashi T., Spodnik J.H. (2000). Structural changes of mitochondria during free radical-induced apoptosis. Folia Morphol. (Warsz).

[69] Ball A.L., Kamalian L., Alfirevic A., Lyon J.J., Chadwick A.E. (2016). Identification of the Additional Mitochondrial Liabilities of 2-Hydroxyflutamide When Compared with its Parent Compound, Flutamide in HepG2 Cells. Toxicol. Sci..

[70] Wang D., Green M.F., McDonnell E., Hirschey M.D. (2013). Oxygen flux analysis to understand the biological function of sirtuins. Methods Mol. Biol..

[71] Brand M.D., Nicholls D.G. (2011). Assessing mitochondrial dysfunction in cells. Biochem. J..

[72] Ming L., Byrne N.M., Camac S.N., Mitchell C.A., Ward C., Waugh D.J., McKeown S.R., Worthington J. (2013). Androgen deprivation results in time-dependent hypoxia in LNCaP prostate tumours: Informed scheduling of the bioreductive drug AQ4N improves treatment response. Int. J. Cancer.

[73] Pignatta S., Arienti C., Zoli W., Di Donato M., Castoria G., Gabucci E., Casadio V., Falconi M., De Giorgi U., Silvestrini R. (2014). Prolonged exposure to (R)-bicalutamide generates a LNCaP subclone with alteration of mitochondrial genome. Mol. Cell Endocrinol..

[74] Li F., Mahato R.I. (2014). MicroRNAs and drug resistance in prostate cancers. Mol. Pharm..

[75] Delgado-Enciso I., Soriano-Hernandez A.D., Rodriguez-Hernandez A., Galvan-Salazar H.R., Montes-Galindo D.A., Martinez-Martinez R., Valdez-Velazquez L.L., Gonzalez-Alvarez R., Espinoza-Gomez F., Newton-Sanchez O.A. (2015). Histological changes caused by meclofenamic acid in androgen-independent prostate cancer tumors: Evaluation in a mouse model. Int. Braz. J. Urol..

[76] D’Angelo G., Duplan E., Boyer N., Vigne P., Frelin C. (2003). Hypoxia up-regulates prolyl hydroxylase activity: A feedback mechanism that limits HIF-1 responses during reoxygenation. J. Biol. Chem..

[77] Luo W., Zhong J., Chang R., Hu H., Pandey A., Semenza G.L. (2010). Hsp70 and CHIP selectively mediate ubiquitination and degradation of hypoxia-inducible factor (HIF)-1alpha but Not HIF-2alpha. J. Biol. Chem..

[78] Zhou Z.L., Luo Z.G., Yu B., Jiang Y., Chen Y., Feng J.M., Dai M., Tong L.J., Li Z., Li Y.C. (2010). Increased accumulation of hypoxia-inducible factor-1alpha with reduced transcriptional activity mediates the antitumor effect of triptolide. Mol. Cancer.

[79] Wang S., Shao X., Li X., Su X., Huo Y., Yang C. (2016). HIF-1alpha may provide only short-term protection against ischemia-reperfusion injury in Sprague-Dawley myocardial cultures. Mol. Clin. Oncol..

[80] Malhotra R., Tyson D.W., Rosevear H.M., Brosius F.C. (2008). Hypoxia-inducible factor-1alpha is a critical mediator of hypoxia induced apoptosis in cardiac H9c2 and kidney epithelial HK-2 cells. BMC Cardiovasc. Disord..

[81] Mao S., Huang S. (2013). The signaling pathway of hypoxia inducible factor and its role in renal diseases. J. Recept. Signal Transduct. Res..

[82] Chen G., Ray R., Dubik D., Shi L., Cizeau J., Bleackley R.C., Saxena S., Gietz R.D., Greenberg A.H. (1997). The E1B 19K/Bcl-2-binding protein Nip3 is a dimeric mitochondrial protein that activates apoptosis. J. Exp. Med..

[83] Yang Y., Yu X., Zhang Y., Ding G., Zhu C., Huang S., Jia Z., Zhang A. (2018). Hypoxia-inducible factor prolyl hydroxylase inhibitor roxadustat (FG-4592) protects against cisplatin-induced acute kidney injury. Clin. Sci. (Lond.).

[84] Cianciolo G., De Pascalis A., Di Lullo L., Ronco C., Zannini C., La Manna G. (2017). Folic Acid and Homocysteine in Chronic Kidney Disease and Cardiovascular Disease Progression: Which Comes First?. Cardiorenal Med..

[85] Owusu-Ansah E., Yavari A., Banerjee U. (2008). A protocol for in vivo detection of reactive oxygen species. Protoc. Exch..

[86] Foresti R., Bucolo C., Platania C.M., Drago F., Dubois-Rande J.L., Motterlini R. (2015). Nrf2 activators modulate oxidative stress responses and bioenergetic profiles of human retinal epithelial cells cultured in normal or high glucose conditions. Pharmacol. Res..

[87] Livak K.J., Schmittgen T.D. (2001). Analysis of relative gene expression data using real-time quantitative PCR and the 2(-Delta Delta C(T)) Method. Methods.

[88] Hsieh C.L., Peng C.C., Chen K.C., Peng R.Y. (2013). Rutin (quercetin rutinoside) induced protein-energy malnutrition in chronic kidney disease, but quercetin acted beneficially. J. Agric. Food Chem..

