# Supplementary material for: Bicalutamide Elicits Renal Damage by Causing Mitochondrial Dysfunction via ROS Damage and Upregulation of HIF-1α
Source: Int J Mol Sci. 2020 May 11;21(9):3400. doi: 10.3390/ijms21093400 (PMC7247665; doi:10.3390/ijms21093400)
Supplement: Supplementary file 1 [file ijms-21-03400-s001.pdf]

Supplementary

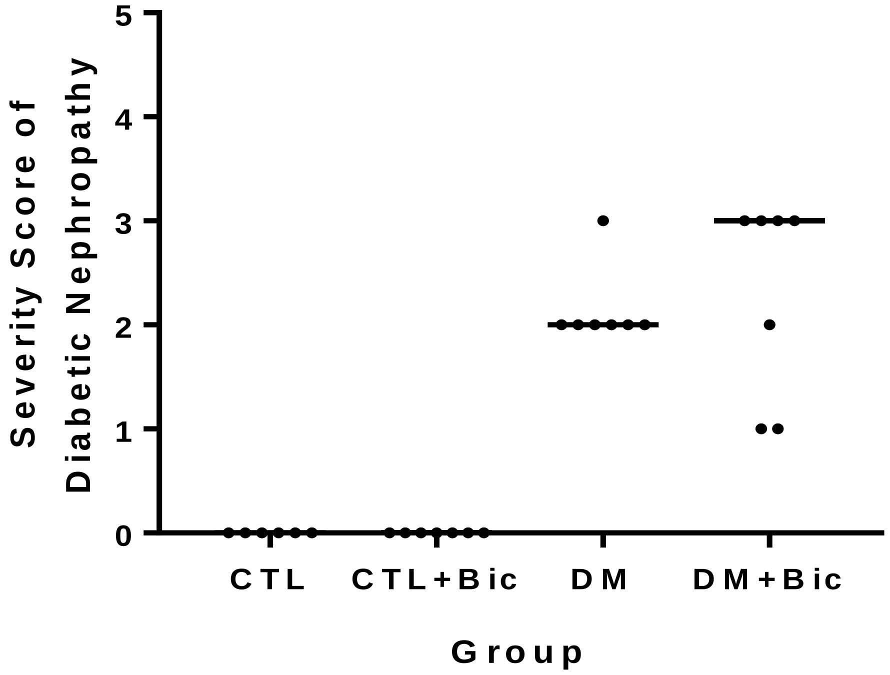

Figure 1. Cluster plot of the pathological score of in vivo experimental rats with diabetic nephropathy. Although bicalutamide (Bic) elicited no influence in the normal control (CTL) +Bic-treated groups, on the contrary, Bic obviously showed more-severe pathological changes in DM+Bic subjects compared to the DM group. Hence, we performed an in vitro experiment with the rat mesangial cell (RMC) line cultured in high-glucose medium to mimic such a DM condition. (CTL, control; CTL+Bic,control and bicalutamide;Bic, bicalutamide; DM+Bic, diabetes and bicalutamide) (Published in the Supplementary information of Sci Rep 9, 3392 (2019) by Peng et al., the image is licensed under a Creative Commons Attribution 4.0 International License.)

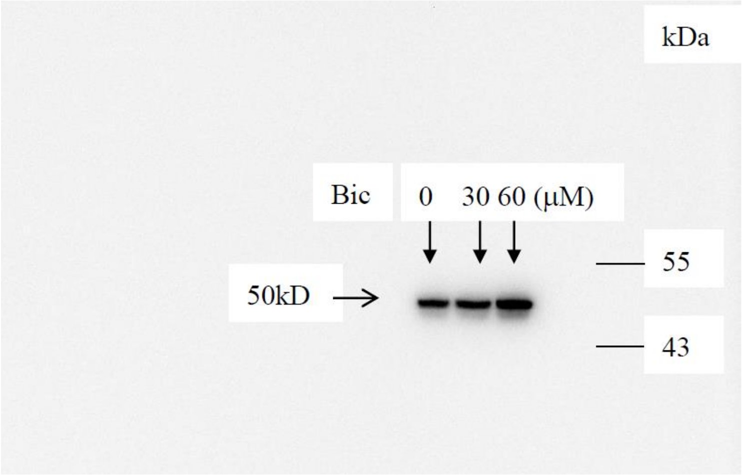

KIM-1

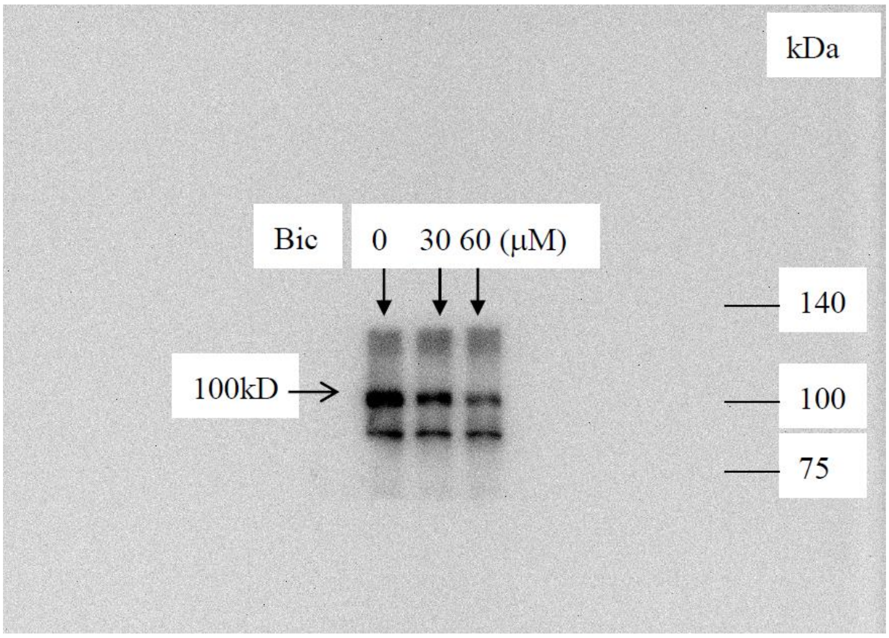

N-cadherin

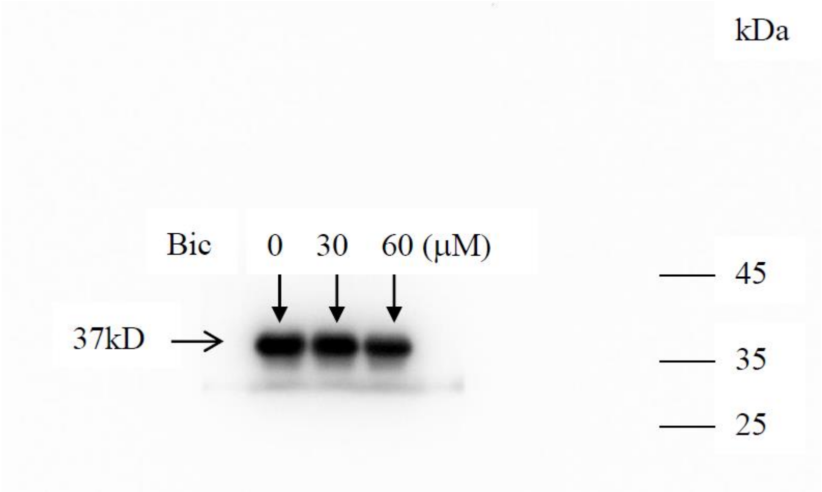

GAPDH

Figure 2. Original Western blot for Fig. 1b. Protein expressions of kidney injury molecule (KIM)-1, N-cadherin, and GAPDH in rat mesangial cells (RMCs).

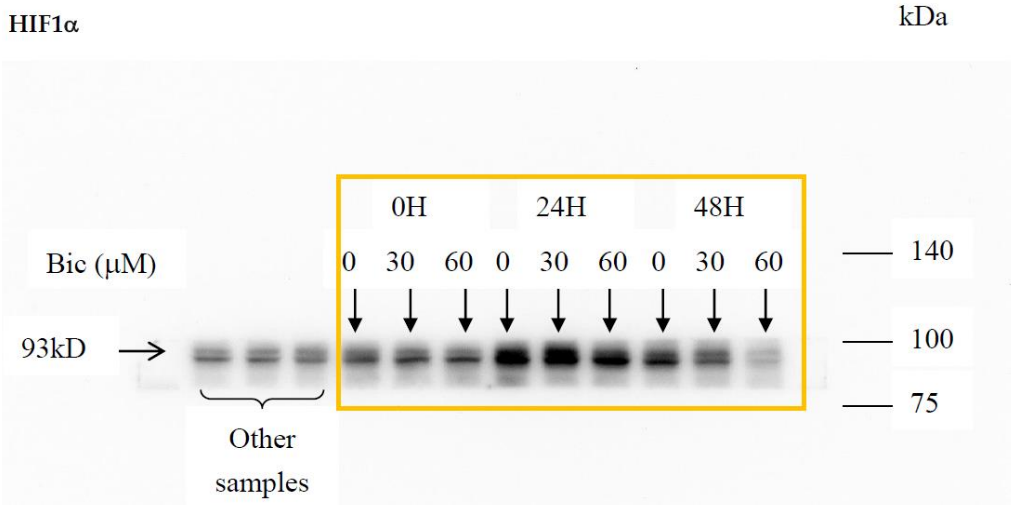

HIF1α

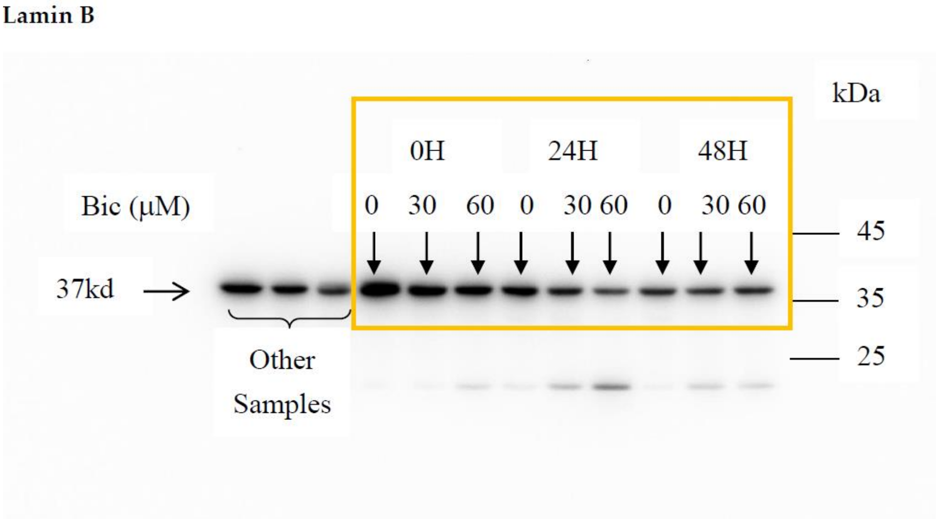

Lamin β

Figure 3. Original Western blot for Fig. 5c. Protein expressions of hypoxia-inducible factor (HIF)-1α and lamin β in rat mesangial cells (RMCs).

BNIP3

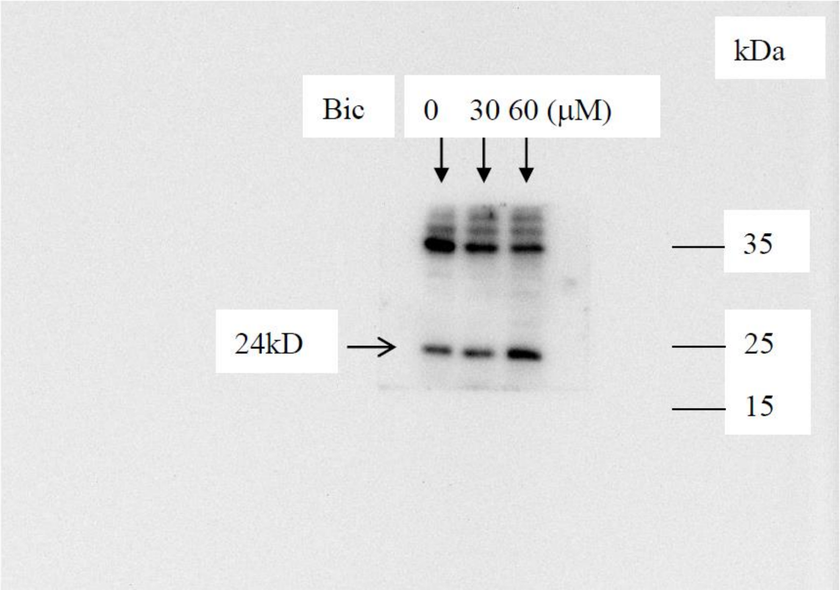

BNIP3

β-actin

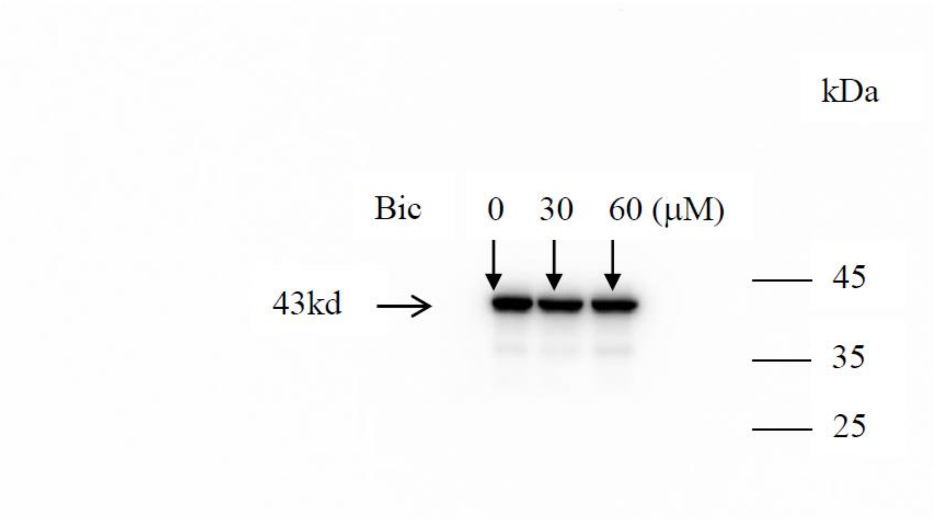

β-actin

Figure 4. Original Western blot for Fig. 6a. Protein expression of Bcl2-interacting protein 3 (BNIP3) and β-actin in rat mesangial cells (RMCs).

Caspase 3

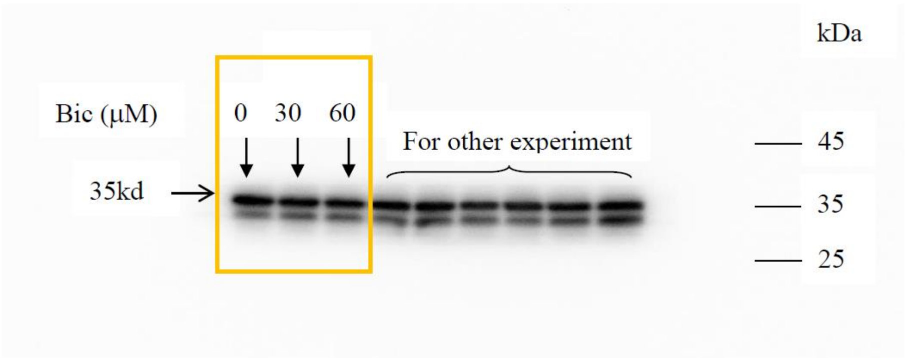

caspase-3

Cleaved caspase 3

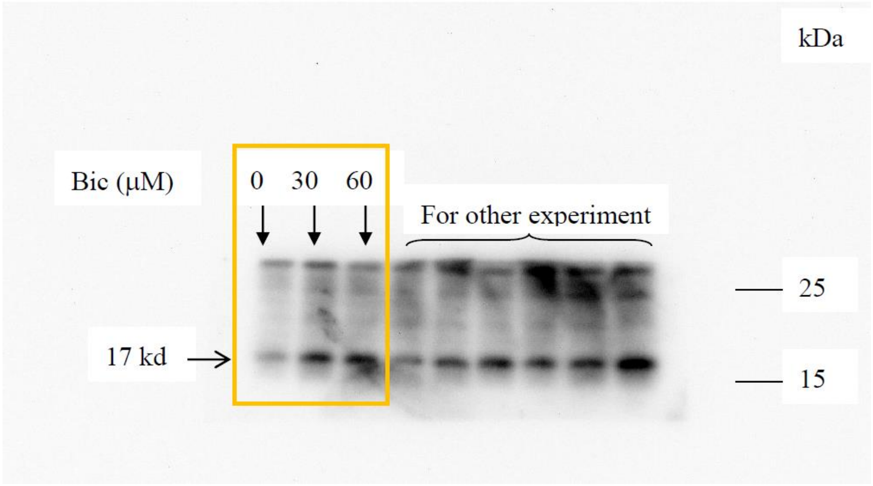

cleaved caspase-3

β-actin

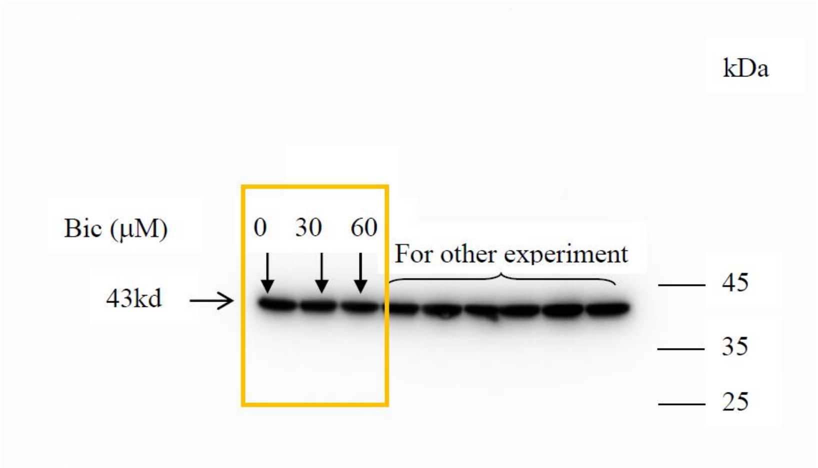

β-actin

Figure 5. Original Western blot for Fig.6a. Protein expressions of caspase-3, cleaved caspase-3, and β-actin in rat mesangial cells (RMCs).
